# Supplementary material for: Coutilisation of oral rehydration solution and zinc for treating diarrhoea and its associated factors among under-five children in East Africa: a multilevel robust Poisson regression
Source: BMJ Open. 2024 Mar 7;14(3):e079618. doi: 10.1136/bmjopen-2023-079618 (PMC10921486; doi:10.1136/bmjopen-2023-079618)
Supplement: Author's manuscript [file bmjopen-2023-079618.draft_revisions.pdf]

BMJ Open

BMJ Open is committed to open peer review. As part of this commitment we make the peer review history of every article we publish publicly available.

When an article is published we post the peer reviewers' comments and the authors' responses online. We also post the versions of the paper that were used during peer review. These are the versions that the peer review comments apply to.

The versions of the paper that follow are the versions that were submitted during the peer review process. They are not the versions of record or the final published versions. They should not be cited or distributed as the published version of this manuscript.

BMJ Open is an open access journal and the full, final, typeset and author-corrected version of record of the manuscript is available on our site with no access controls, subscription charges or pay-per-view fees (http://bmjopen.bmj.com).

If you have any questions on BMJ Open's open peer review process please email info.bmjopen@bmj.com

Co-utilization of oral rehydration solution and zinc for treating diarrhea, and its associated factor among under-five children in East Africa: a multilevel robust poisson regeression.

Journal:	*BMJ Open*
Manuscript ID	bmjopen-2023-079618
Article Type:	Original research
Date Submitted by the Author:	07-Sep-2023
Complete List of Authors:	Legesse, Bruck Tesfaye; Wollega University, Department of Pediatrics and Neonatal Nursing<br>Wondie, Wubet ; Ambo University College of Medicine and Public Health, Department of Pediatrics and Child Health Nursing; Ambo University, Department of Pediatrics and Child Health Nursing<br>Gedefaw, Gezahagn ; University of Gondar College of Medicine and Health Sciences, Department of Neonatal Health Nursing<br>Workineh, Yakob Tadese; Wolaita Sodo University, Department of Pediatrics and Child Health Nursing<br>Seifu, Beminate; Samara University, Public health
Keywords:	PREVENTIVE MEDICINE, Community child health < PAEDIATRICS, Microbiology < PATHOLOGY

SCHOLARONE™
Manuscripts

I, the Submitting Author has the right to grant and does grant on behalf of all authors of the Work (as defined in the below author licence), an exclusive licence and/or a non-exclusive licence for contributions from authors who are: i) UK Crown employees; ii) where BMJ has agreed a CC-BY licence shall apply, and/or iii) in accordance with the terms applicable for US Federal Government officers or employees acting as part of their official duties; on a worldwide, perpetual, irrevocable, royalty-free basis to BMJ Publishing Group Ltd ("BMJ") its licensees and where the relevant Journal is co-owned by BMJ to the co-owners of the Journal, to publish the Work in this journal and any other BMJ products and to exploit all rights, as set out in our licence.

The Submitting Author accepts and understands that any supply made under these terms is made by BMJ to the Submitting Author unless you are acting as an employee on behalf of your employer or a postgraduate student of an affiliated institution which is paying any applicable article publishing charge ("APC") for Open Access articles. Where the Submitting Author wishes to make the Work available on an Open Access basis (and intends to pay the relevant APC), the terms of reuse of such Open Access shall be governed by a Creative Commons licence – details of these licences and which Creative Commons licence will apply to this Work are set out in our licence referred to above.

Other than as permitted in any relevant BMJ Author's Self Archiving Policies, I confirm this Work has not been accepted for publication elsewhere, is not being considered for publication elsewhere and does not duplicate material already published. I confirm all authors consent to publication of this Work and authorise the granting of this licence.

For peer review only

Co-utilization of oral rehydration solution and zinc for treating diarrhea, and its associated factor among under-five children in East Africa: a multilevel robust poisson regeression.

Bruck Tesfaye Legesse[1]*, Wubet Tazeb Wondie[2] Gezahagn Demsu Gedefaw[3], Yakob Tadese Workineh[4], Beminate Lemma Seifu[5],

Affiliations

[1] Department of Pediatrics and Neonatal Nursing, School of Nursing and Midwifery, Institute of Health Sciences, Wollega University, Nekemte, Ethiopia.

[2]Department of Pediatrics and Child Health Nursing, College of Medicine and Health Sciences, Ambo University, Ambo, Ethiopia

[3]Department of Neonatal Health Nursing, School of Nursing, College of Medicine and Health Sciences, and Specialized Hospital, University of Gondar, Gondar, Northwest Ethiopia

[4]Department of Pediatrics and Child Health Nursing, School of Nursing, College of Medicine and Health Sciences, Wolaita sodo University, Wolaita sodo, Ethiopia.

[5]Department of Public Health, College of Medicine and Health Sciences, Samara University, Samara, Ethiopia.

***Corresponding author:** Bruck Tesfaye Legesse; Email: **brucktesfaye143@gmail.com**

For peer review only

Abstract

Background: Childhood diarrhoea continues to be the most significant cause of death among children under five globally, despite the fact that it can be cured with an affordable treatment. Oral rehydration salts and zinc are seldom used to treat diarrhoea in contexts of East Africa. The aim of this study was to assess the Co-utilization of oral rehydration solution and zinc for treating diarrhea, and its associated factor among under-five children in East Africa.

Methods: Recent Demographic and Health Survey (DHS) data from 12 East African countries were used and a total of 16,850 participants was included in the study. A generalized linear mixed model with robust error variance was used to account for the clustering effects of the DHS data and the binary nature of the outcome variable. An adjusted prevalence ratio with a 95% confidence interval (CI) was reported to declare the statistical significance.

Result: In East African nations, the co-utilization of ORS and zinc for the treatment of diarrhoea in children under five was 53.27% with a 95% CI (52.54%, 54.01%). Children of mothers with primary education (aPR = 1.15, 95% CI = 1.09, 1.20), secondary education (aPR=1.08, 95% CI= 1.02-1.14), higer education (aPR=1.19, 95% CI= 1.10-1.29), those from maternal age category of 20-24 (aPR=1.14, 95% CI= 1.07-1.21), age category of 25-29 (aPR=1.13, 95% CI= 1.06-1.21), age category of 30-34 (aPR=1.09, 95% CI= 1.02-1.16), those from wealthy households (aPR = 1.04, 95% CI = 1.01, 1.09), and those who have a media exposure (aPR=1.04, 95% CI= 1.01-1.08) were more likely to receive combination.

Conclusion: Only half of under-five children with diarrhea in east Africa were treated with a combination of ORS and zinc. To increase the use of the suggested combination therapy of ORS with zinc, it is importance to empower women through education, prevent teen pregnancy.

For peer review only

Keywords: Associated factors;Diarrhea; East Africa: multilevel analysis; ORS and Zinc co-utilization; Under five children

Strength and limitation

This study was based on nationally representative data and used appropriate statistical analysis techniques. This is also the first study to look at the factors that influence ORS and zinc co-utilization in children under the age of five in East Africa. Despite the aforementioned strengths, the measure of zinc utilization practice was based on the mother's recall, which could lead to recall bias. Second, the DHS survey lacks information on zinc availability and the role of diet in zinc intake. As a result, we are unable to evaluate these factors. Furthermore, due to the cross-sectional nature of the data, a clear temporality (cause and effect relationship) between the dependent and independent variables was not established.

Background

Diarrhea is three or more loose or watery stools in a 24-hour period, which can also present as a change in the volume, frequency, or fluidity of faeces (1). Although there are easy ways to prevent and cure diarrhoea in children, it continues to be the world's greatest cause of mortality and undernutrition in children under the age of five (2). Each year, around 525,000 children under the age of five lose their lives to diarrhoea (3), and 10 countries in Asia and Africa: Bangladesh, Democratic Republic of the Congo, Ethiopia, India, Kenya, Niger, Nigeria, Pakistan, Tanzania, and Uganda accounts for 60% of these fatalities. Low and middle-income countries (LMICs) have a high fatality rate from diarrhoea because the condition is common there and is linked to high-risk factors such malnutrition, contaminated water sources, and a lack of access to necessary care. (2).

For peer review only

One of the top priorities for reducing unnecessary child deaths under the Sustainable Development Goal is treating diarrhoea (4). Up to 93% of diarrhea-related death can be avoided using ORS and zinc, two very affordable and extremely effective diarrhoea therapies. Because of this, the World Health Organisation (WHO) and the United Nations Children's Fund (UNICEF) suggest combining ORS with zinc for a successful therapy (5, 6). While zinc supplementation lessens the length and intensity of episodes and lowers the likelihood of recurrence in the short term, ORS replenishes the vital fluids and salts lost via diarrhoea. However, the global prevalence of using ORS with zinc to treat diarrhoea continues to be alarmingly low (2). Less than 7% of children under the age of five in LMICs receive zinc treatment, according to a 2017 UNICEF report, and only 44% of them receive ORS. Access to this life-saving medicine might be made easier in many of these nations, many of which have high mortality rates from diarrhoea(7) .

In East Africa, only a small percentage of under five children receive effective treatment or ORS with Zinc for diarrheal disease. As study in Kenya mentioned that childrens treated with bundled ORS with zinc accounts 15% (8), while other study in Ethiopia found only 16.65% (9). Other study conducted in Uganda mentioned that 30% of those under five children treated for diarrhea took co-packed ORS with zinc (10).

According to studies done so far maternal education, economic status, media exposure, residency, proximity to a medical facility, and health insurance coverage have all been found to influence the combination of ORS and zinc for the treatment of diarrhoea in children under the age of five (9, 11-13).

Despite the clinical significance of using ORS and zinc together, there is limited evidence no research that ascertain the prevalence and contributing variables of ORS and zinc co-use among young children with diarrhoea in East Africa. This investigation was thus carried out to close this

For peer review only

gap. As this study is the first to its type in East Africa it may also be used as a baseline by the government and policymakers to take actions to increase its uptake.

Methods

Data source, study setting, and population

This study was based on the most recent Demographic and Health Survey (DHS) data of 12 east African countries. DHS is a nationally representative survey routinely conducted every five years and collects data on basic health indicators like mortality, morbidity, fertility, and maternal and child health-related characteristics. The survey used a two-stage stratified sampling technique to select the study participants. In the first stage, Enumeration Areas (EAs) were randomly selected based on the country's recent population, and using the housing census as a sampling frame, households were randomly selected in the second stage. Each country's survey consists of different datasets including men, women, children, birth, and household datasets. For this study, the study population was under five children, thus, we used the kid's record dataset (KR file). The source population of this study was all under-five children with diarrhea in east Africa. The study population was all under-five children with diarrhea within the last two weeks preceding the survey. In the current study, a weighted sample of 16,850 under-five children was considered for final analysis. Details about DHS methodology can be accessed at https://dhsprogram.com/Methodology/index.cfm.

Study variables and definitions

Dependent variable: The co-utilization of ORS and zinc was dichotomized as "yes=1" if the child uses both ORS and zinc for the treatment of childhood diarrhea and "no=0" if the child does not use both ORS and zinc for the treatment of childhood diarrhea.

For peer review only

Independent variables: The independent variables were classified as a community and individual-level variables. Individual-level factors were maternal age, educational status of the mother, father's educational status, working status of the mother, wealth status, media exposure, sex of household head, health insurance, sex of the child. Distance to health facility, place of residence (urban or rural), community women's education and community poverty level were considered as community-level factors.

Media exposure: was created from three variables (frequency of listening to the radio, watching television, and reading newspapers or magazines). In this study, women who listened to radio or watched television, or read newspapers/magazines at least less than once a week were considered as having exposure to media (coded "Yes") and otherwise labeled as not having media exposure (coded "No").

Community women's education: defined as the proportion of women who attended primary, secondary, and higher education within the cluster. The aggregate of individual women's primary, secondary, and higher educational attainment can show the overall educational status of women within the cluster. They were categorized into two categories as a higher proportion of women's education within the cluster and a lower proportion of women's education based on the national median value.

Community poverty status: defined as the proportion of poor and poorest mothers within the cluster. For each cluster proportion of poor and poorest was aggregated and show overall poverty status within the cluster. It was categorized into two categories based on the national median value a higher proportion of poor/poorest mothers and a lower proportion of mothers within a cluster.

For peer review only

Data management and statistical analysis

Data extraction, coding, and analysis were done using Stata version 17 statistical software. The weighted data were used for analysis to restore the representativeness of the data. Since the DHS data has a hierarchical nature, the Intra-class Correlation Coefficient (ICC) was estimated to assess the clustering effect. The ICC indicated that there was a significant clustering effect (ICC>10%). This study was a cross-sectional study and the prevalence of co-utilization of ORS and zinc was greater than 10%, and if we report the odds ratio, it could overestimate the association between co-utilization of ORS and zinc and the independent variables. In such cases, the prevalence ratio is the best measure of association, and therefore, multilevel Poisson regression analysis with robust variance was fitted to identify predictors of co-utilization of ORS and zinc among under-five children. Variables with a p-value<0.2 in the bi-variable multilevel Poisson regression analysis were considered for the multivariable analysis.

Four models were constructed for the multilevel Poisson regression analysis. The first model was a null model without explanatory variables to determine the extent of cluster variation in the co-utilization of ORS and zinc. The second model was fitted with individual-level variables, the third with community-level variables, and the fourth with both individual and community-level variables at the same time. Because the models were nested, the deviance (-2Log-Likelihood Ratio (LLR)) was used to compare them, and the model with the lowest deviance was the best-fitted model for the data. Finally, the adjusted prevalence ratio (APR) with its 95% confidence interval (CI) was reported, and variables with p value<0.05 in the multivariable analysis were considered as significant predictors of co-utilization of ORS and zinc among under-five children.

For peer review only

Ethical consideration

Permission to access the data used in this study was obtained from a measure demographic and health survey through an online request from http://www.dhsprogram.com. The data are publicly available from the program's official database.

Results

Socio-demographic characteristics

A total weighted sample of 16,850 under-five children who had diarrhea within 2 weeks preceding the survey were included in this study. More than half, 8,965 (53.20%) of the children were males while the vast majority (79.10%) of them resided in rural areas. The majorities, 7,778 (46.16%) were from poor households and 3,666 (21.76%) had mothers who did not have a formal education. Only 818 (5.72%) were covered by health insurance (Table 1).

Table 1:: Socio-demographic and economic characteristics of study participants east Africa

Variable	Weighted frequency	Percent
**Individual level variables**		
**Sex of the child**		
Male	8,965	53.20
Female	7,885	46.80
**Age of the mother**		
15-19	1,433	8.51
20-24	4,715	27.98
25-29	4,545	26.97
30-34	3,228	19.16
35-39	1,965	11.66
40-44	757	4.49
45-49	206	1.22
**Marital status**		
Never in union	965	5.73
Married/ living together	14,160	84.03
Divorced / widowed/ separated	1,725	10.24

For peer review only

**Maternal education**		
No formal education	3,666	21.76
Primary	8,544	50.71
Secondary	3,942	23.40
Higher	697	4.14
**Father's education (n=14,164)**		
No formal education	2,667	18.83
Primary	6,842	48.31
Secondary	3,657	25.82
Higher	997	7.04
**Maternal current working status**		
Not Employed	5,747	34.11
Employed	11,103	65.89
**Sex of household head**		75.84
Male	12,778	24.16
Female	4,072	
**Household wealth status**		
Poor	7,778	46.16
Middle	3,218	19.10
Rich	5,854	34.74
**Covered by health insurance (n=14,291)**		
No	13,472	94.28
Yes	818	5.72
**Media exposure**		
No	5,959	35.36
Yes	10,891	64.64
**Community level variables**		
**Distance to health facility (n=15,498)**		
Big problem	6,926	44.69
Not a big problem	8,572	55.31
**Residence**		
Urban	3,521	20.90
Rural	13,329	79.10
**Community educational status**		
Low	2,996	17.78
High	13,854	82.22
**Community poverty level**		
Low	7,695	45.67
Hgh	9,155	54.33

For peer review only

The prevalence of ORS and Zinc co-utilization for diarrhea treatment

The pooled prevalence of co-utilization of ORS and zinc for the treatment of diarrhea among under five children in East African countries was 53.27% with a 95% CI (52.54%, 54.01%). In East African countries, the prevalence of ORS and zinc co-utilization ranges from 24.36% (95% CI: 21.85, 26.86) in Madagascar to 69.32% (95% CI: 67.76, 70.87) in Malawi (Figure 1).

Figure 1: Forest plot of pooled prevalence of co-utilization of ORS and zinc for the treatment of diarrhea among under-five children in East African countries

Random effect and model comparison

To evaluate the random or clustering effect using the intraclass correlation coefficient (ICC) and we used deviance for model comparison. The ICC in the null model was 0.145, indicating that about 14.5% of the total variation in co-utilization of ORS, zinc was attributable to unmeasured or unmeasurable factors (random effects). Regarding model comparison, the final model (a model with lower deviance) was the best-fitted model (Table 2).

Table 2: Random effect and model comparison for factors associated with co-utilization of ORS and zinc

	Null model	Model 1	Model 2	Model 3
ICC	14.56%	15.31%	15.01%	15.01%
Log likelihood	-14402.21	-14369.22	-14398.24	-14367.59
Deviance	28,804.42	28,738.44	-28,796.48	28,735.18
ICC, intraclass correlation coefficient				

Factors associated with co-utilization of ORS and Zinc for diarrhea treatment

A multilevel bi-variable analysis were done to assess the crude relationship between the independent variables and ORS-zinc co-utilization. Among the variables included in the bi-

For peer review only

variable analysis only maternal age, maternal education status, wealth index, maternal employment status, media exposure, community level illiteracy and community level povery showed a statistically significant association with ORS and zinc co-utilization at a *p*-value of < 0.20.

In the final model maternal age, maternal education status, wealth index and media exposure were significantly associated with ORS and zinc co-utilization (p-value N 0.05). The prevalence of ORS and zinc co-utilization was 1.14 (APR=1.14, 95%CI: 1.07, 1.21), 1.13 (APR=1.13, 95%CI: 1.06, 1.21) and 1.09 (APR=1.09, 95%CI: 1.02, 1.16) times higher in children whose mothers were aged 20-24, 25-29, and 30-34, respectively. Being the child of a woman with a primary, secondary, or higher level of education raises the prevalence of co-utilization by 1.15, 1.08, and 1.19, respectively. Compared to children from poor household wealth index, those children from rich wealth index had 4 % higher prevalence of ORS and zinc co-utilization. The prevalence of ORS and zinc co-utilization among children whose mothers had media exposure was 1.04 times higher as compared to their counterparts (APR = 1.04, 95% CI :1.01, 1.08) (Table 3).

Table 3:Factors associated with co-utilization of ORS and zinc for management of diarrhea among under-five children

**Variable**	**ORS and Zinc Co-utilization**		**CPR (95%CI)**	**APR (95%CI)**
**Individual level variables**	**No (%)**	**Yes (%)**		
**Age of the mother**				
15-19	746 (52.03)	687 (47.97)	1	1
20-24	2,106 (44.67)	2,609 (55.33)	1.13 (1.06, 1.19)	1.14 (1.07, 1.21)*
25-29	2,026 (44.57)	2,519 (55.43)	1.12 (1.05, 1.19)	1.13 (1.06,1.21)*
30-34	1,573 (48.73)	1,654 (51.27)	1.06 (0.99, 1.13)	1.09 (1.02, 1.16)*
35-39	982 (49.99)	982 (50.01)	1.01 (0.94, 1.09)	1.04 (0.97, 1.12)
40-44	400 (52.90)	357 (47.10)	0.95 (0.87, 1.04)	1.00 (0.92, 1.09)
45-49	106 (51.32)	100 (48.68)	0.98 (0.85, 1.13)	1.05 (0.91, 1.21)

For peer review only

**Maternal educational status**				
No formal education	2,004 (54.67)	1,662 (45.33)	1	1
Primary	3,873 (45.33)	4,671 (54.67)	1.17 (1.12, 1.23)	1.15 (1.09, 1.20)*
Secondary	1,806 (45.81)	2,136 (54.19)	1.14 (1.08, 1.19)	1.08 (1.02, 1.14)*
Higher	256 (36.75)	440 (63.25)	1.28 (1.19, 1.38)	1.19 (1.10, 1.29)*
**Household wealth index**				
Poor	3,819 (49.10)	3,958 (50.90)	1	1
Middle	1,511 (46.96)	1,707 (53.04)	1.05 (1.01, 1.09)	1.03 (0.99, 1.08)
Rich	2,609 (44.57)	3,245 (55.43)	1.07 (1.03, 1.12)	1.04 (1.01, 1.09)*
**Maternal employment status**				
Not employed	2,769 (48.19)	2,977 (51.81)	1	1
Employed	5,170 (46.57)	5,933 (53.43)	0.98 (0.95, 1.02)	0.97 (0.94, 1.04)
**Media exposure**				
No	3,022 (50.72)	2,936 (49.28)	1	1
Yes	4,917 (45.15)	5,974 (54.85)	1.08 (1.04, 1.12)	1.04 (1.01, 1.08)*
**Community illiteracy level**				
Low	1,287 (42.96)	1,709 (57.04)	1	1
High	6,653 (48.02)	7,201 (51.98)	0.92 (0.89, 0.96)	0.96 (0.92, 1.03)
**Community poverty level**				
Low	3,641 (47.32)	4,053 (52.68)	1	1
High	4,298 (46.95)	4,857 (53.05)	0.99 (0.97, 1.03)	1.03 (0.99, 1.07)

*p-value N0.05, CPR: Crude Prevalence Ratio, APR: Adjusted Prevalence Ratio

Discussion

The aim of this study was to assess the factors associated with the co-use of ORS and zinc for the treatment of diarrhoea in children under five years old across East African countries. In our study, the co-use of ORS and zinc was associated with the mother's age, her educational level, family's financial status, and media exposure.

This study mentioned maternal age above twenty is associated with co-utilization of ORS and zinc and this finding is supported by different study conducted in Nigeria (14). The possible reason for this finding is older mothers were more likely than younger mothers to be knowledgeable about

For peer review only

paediatric diarrhoea and how to treat it and Compared to older mothers, younger mothers do not have as much exposure. This study found that maternal educational status was associated with usage of ORS with zinc to treat under five diarrheal disease and this finding is supported with study conducted in Pakistan (15), India (16), Nigeria (11), Cameron (17), Kenya (18), and Ethiopia (9, 13, 19-21). This may be because of educated women are more likely to take sick children to the medical facility and a woman with education is more likely than a mother without education to take her ill kid for follow-up as well as to take the ordered medication as per recommended. In addition to the above reason, educated mother have a good awareness regarding the the health problems of their kid than a a mother without education (22, 23).

Our study mentioned that media exposure was one of the factors associated with diarrheal treatment using ORS with zinc. This finding is inline with study conducted in India (16), Bangladesh (24), Ghana (25), and Ethiopia (13, 21). This may be due to the media's important role in increasing the mother's or caregiver's understanding and awareness of the best ways to treat diarrhoea in children (24).

As this study finding, family's wealth status affects the usage of ORS with zinc. This finding is supported by different studies conducted in India (26), and Ethiopia (13). This is due to the fact that low-income households in low and middle-income countries (LMICs), such as those in Africa, particularly in East Africa, cannot afford the expenses of healthcare services, including ORS and zinc. Thus, when children from low-income households get diarrhoea, they might not receive ORS with zinc (27).

For peer review only

Conclusion

In this study, media exposure, rich household wealth status, maternal education, and maternal age over twenty were associated factors for ORS and zinc co-use. In order to raise theprevalence of ORS and zinc co-utilization for the successful treatment of diarrhoea in children under the age of five, it is advised to increase media exposure, maternal education, and support household wealth index. In addition, policies and intervention programmes that aim to stop unnecessary deaths of children under the age of five should be prioritised by preventing adolescent pregnancy.

Abbreviations: aPR, Adjusted Prevalence Ratio; CI, Confidence Interval; uPR, Unadjusted Prevalence Ratio; DHS, Demographic and Health Survey; ICC, Intraclass Correlation Coefficient; LL: Log-likelihood; UNICEF: United Nations Children's Fund

Competing interests

The authors declare that they have no competing interests.

Authors' contributions

Conceptualization: Bruck Tesfaye Legesse, Beminate Lemma Seifu

Data curation: Bruck Tesfaye Legesse, Beminate Lemma Seifu

Formal analysis: Beminate Lemma Seifu, Bruck Tesfaye Legesse

Investigation: Bruck Tesfaye Legesse, Beminate Lemma Seifu

Methodology: Beminate Lemma Seifu, Bruck Tesfaye Legesse

Software: Bruck Tesfaye Legesse, Beminate Lemma Seifu

For peer review only

Validation: Beminate Lemma Seifu, Bruck Tesfaye Legesse, Wubet Tazeb Wondie, Gezahagn Demsu Gedefaw, Yakob Tadese Workineh

Writing – original draft: Bruck Tesfaye Legesse, Beminate Lemma Seifu

Writing – review & editing: Beminate Lemma Seifu, Bruck Tesfaye Legesse, Wubet Tazeb Wondie, Gezahagn Demsu Gedefaw, Yakob Tadese Workineh

Acknowledgments

We would like to thank the MEASURE DHS for providing the dataset for the study.

Funding

No fund was received for this work.

Availability of data and materials

All the data used for the study are available on a reasonable request.

Consent for publication

Not applicable.

Reference

1. Guarino A, Dupont C, Gorelov AV, Gottrand F, Lee JK, Lin Z, et al. The management of acute diarrhea in children in developed and developing areas: from evidence base to clinical practice. 2012;13(1):17-26.

2. Almasi A, Zangeneh A, Ziapour A, Saeidi S, Teimouri R, Ahmadi T, et al. Investigating Global Spatial Patterns of Diarrhea-Related Mortality in Children Under Five. 2022;10:861629.

For peer review only

3. Mokomane M, Kasvosve I, Melo Ed, Pernica JM, Goldfarb DMJTaiid. The global problem of childhood diarrhoeal diseases: emerging strategies in prevention and management. 2018;5(1):29-43.

4. Lee BX, Kjaerulf F, Turner S, Cohen L, Donnelly PD, Muggah R, et al. Transforming our world: implementing the 2030 agenda through sustainable development goal indicators. 2016;37:13-31.

5. Qazi S, Aboubaker S, MacLean R, Fontaine O, Mantel C, Goodman T, et al. Ending preventable child deaths from pneumonia and diarrhoea by 2025. Development of the integrated Global Action Plan for the Prevention and Control of Pneumonia and Diarrhoea. 2015;100(Suppl 1):S23-S8.

6. Black R, Fontaine O, Lamberti L, Bhan M, Huicho L, El Arifeen S, et al. Drivers of the reduction in childhood diarrhea mortality 1980-2015 and interventions to eliminate preventable diarrhea deaths by 2030. 2019;9(2).

7. Mohamed SOO, Alawad MOA, Ahmed AAM, Mahmoud AAAJBRN. Access to oral rehydration solution and zinc supplementation for treatment of childhood diarrhoeal diseases in Sudan. 2020;13:1-4.

8. Lam F, Wentworth L, Cherutich P, Migiro S, Abdala K, Musyoka M, et al. An evaluation of a national oral rehydration solution and zinc scale-up program in Kenya between 2011 and 2016. 2019;9(1).

9. Kassa SF, Alemu TG, Techane MA, Wubneh CA, Assimamaw NT, Belay GM, et al. The co-utilization of oral rehydration solution and zinc for treating diarrhea and its associated factors among under-five children in Ethiopia: further analysis of EDHS 2016. 2022:1713-21.

10. Lam F, Kirchhoffer D, Buluma DM, Kabunga L, Wamala-Mucheri PN, Schroder K, et al. A retrospective mixed-methods evaluation of a national ORS and zinc scale-up program in Uganda between 2011 and 2016. 2019;9(1).

11. Gwarzo GJNJoP. Mothers' awareness and use of zinc in under-five children with diarrhoea in North-Western Nigeria. 2018;45(2):81-5.

12. Ajayi DJAoB, Medicine A. Determinants of Oral Rehydration Solution and Zinc Use Among Under-Five Children for The Management of Diarrhea in Abeokuta, Nigeria. 2019;7(1):35–9-–9.

For peer review only

13. Negesse Y, Fetene Abebe G, Addisu A, Setegn Alie M, Alemayehu DJFiPH. The magnitude of oral rehydration salt utilization in diarrhea hot spot regions of Ethiopia and its associated factors among under-five children: A multilevel analysis based on Bayesian approach. 2022;10:960627.

14. Okafor IP, Akinyemi OT, Wika-Kobani BN, Olubodun T, Eze UT. Childhood diarrhoea: a cross-sectional survey on maternal knowledge, hygienic practices and use of oral zinc for home management in a Nigerian community. The Pan African medical journal. 2022;42:123.

15. Duggal MN, Bari A, Mansoor A, Saeed S, Rizwan W, Nayar JJJTJotPMA. Awareness of quantity of ORS and Zinc among mothers of under five children with diarrhoea at a tertiary care Centre Lahore. 2021;71(3):921-4.

16. Lam F, Pro G, Agrawal S, Shastri VD, Wentworth L, Stanley M, et al. Effect of enhanced detailing and mass media on community use of oral rehydration salts and zinc during a scale-up program in Gujarat and Uttar Pradesh. 2019;9(1).

17. Essomba NE, Koum DK, Adiogo D, Ngwe M, Coppieters YJMmj. Use of oral rehydration therapy in the treatment of childhood diarrhoea in Douala, Cameroon. 2015;27(2):60-4.

18. Njeri G, Muriithi MJESJ. Household choice of diarrhea treatments for children under the age of five in Kenya: evidence from The Kenya Demographic and Health Survey 2008-09. 2013;9(6):77-91.

19. Ayele E, Tasew H, Mariye T, Teklay G, Alemayhu T, Mesfin FJM. Zinc utilization and associated factors among under-five children having acute diarrhea in Kebri-dehar Town, Somali Region, Ethiopia-2017. 2020;4(1):15-9.

20. Terefa DR, Shama AT, Kenea AKJBo. Sociodemographic and institutional determinants of zinc bundled with oral rehydration salt utilisation among under-five children with diarrhoeal diseases in East Wallaga zone, western Ethiopia: a community-based cross-sectional study. 2023;13(5):e070203.

21. Teshale AB, Liyew AM, Tesema GAJBPH. Factors associated with zinc utilization for the management of diarrhea in under-five children in Ethiopia. 2020;20:1-7.

22. Adane M, Mengistie B, Mulat W, Kloos H, Medhin GJJoH, Population, Nutrition. Utilization of health facilities and predictors of health-seeking behavior for under-five children

For peer review only

with acute diarrhea in slums of Addis Ababa, Ethiopia: a community-based cross-sectional study. 2017;36(1):1-12.

23. Apuleni G, Jacobs C, Musonda PJFiPH. Predictors of health seeking behaviours for common childhood illnesses in poor resource settings in Zambia, a community cross sectional study. 2021;9:569569.

24. Alam Z, Higuchi M, Sarker MAB, Hamajima NJNjoms. Mass media exposure and childhood diarrhea: a secondary analysis of the 2011 Bangladesh demographic and health survey. 2019;81(1):31.

25. El-Khoury M, Banke K, Sloane PJGHS, Practice. Improved childhood diarrhea treatment practices in Ghana: a pre-post evaluation of a comprehensive private-sector program. 2016;4(2):264-75.

26. Bawankule R, Singh A, Kumar K, Pedgaonkar SJBph. Disposal of children's stools and its association with childhood diarrhea in India. 2017;17:1-9.

27. Braimoh T, Danat I, Abubakar M, Ajeroh O, Stanley M, Wiwa O, et al. Private health care market shaping and changes in inequities in childhood diarrhoea treatment coverage: evidence from the analysis of baseline and endline surveys of an ORS and zinc scale-up program in Nigeria. 2021;20:1-12.

For peer review only

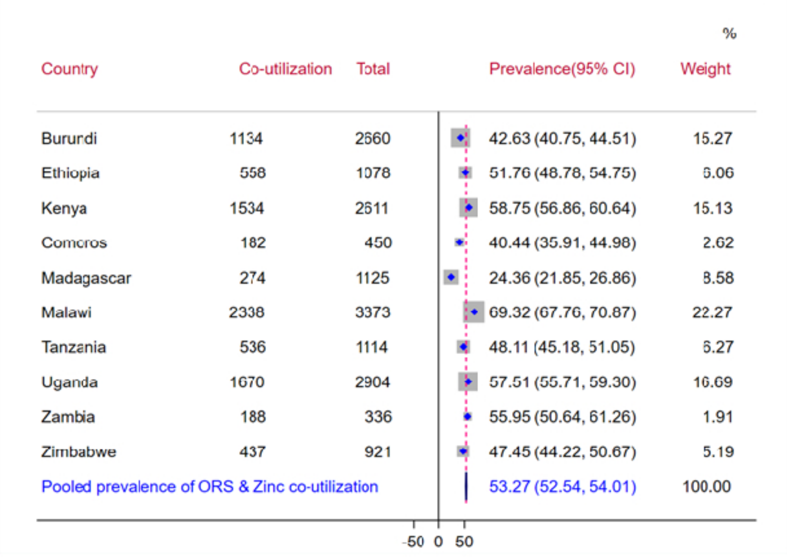

204x120mm (96 x 96 DPI)

STROBE Statement—Checklist of items that should be included in reports of ***cross-sectional studies***

	Item No	Recommendation	Page No
**Title and abstract**	1	(*a*) Indicate the study's design with a commonly used term in the title or the abstract	1
		(*b*) Provide in the abstract an informative and balanced summary of what was done and what was found	2
**Introduction**			
Background/rationale	2	Explain the scientific background and rationale for the investigation being reported	2
Objectives	3	State specific objectives, including any prespecified hypotheses	1, 5
**Methods**			
Study design	4	Present key elements of study design early in the paper	5, 6
Setting	5	Describe the setting, locations, and relevant dates, including periods of recruitment, exposure, follow-up, and data collection	5
Participants	6	(*a*) Give the eligibility criteria, and the sources and methods of selection of participants	5
Variables	7	Clearly define all outcomes, exposures, predictors, potential confounders, and effect modifiers. Give diagnostic criteria, if applicable	6, 7
Data sources/ measurement	8*	For each variable of interest, give sources of data and details of methods of assessment (measurement). Describe comparability of assessment methods if there is more than one group	*7*
Bias	9	Describe any efforts to address potential sources of bias	7
Study size	10	Explain how the study size was arrived at	6, 7
Quantitative variables	11	Explain how quantitative variables were handled in the analyses. If applicable, describe which groupings were chosen and why	
Statistical methods	12	(*a*) Describe all statistical methods, including those used to control for confounding	7
		(*b*) Describe any methods used to examine subgroups and interactions	
		(*c*) Explain how missing data were addressed	
		(*d*) If applicable, describe analytical methods taking account of sampling strategy	
		(*e*) Describe any sensitivity analyses	
**Results**			
Participants	13*	(a) Report numbers of individuals at each stage of study—eg numbers potentially eligible, examined for eligibility, confirmed eligible, included in the study, completing follow-up, and analysed	5
		(b) Give reasons for non-participation at each stage	
		(c) Consider use of a flow diagram	
Descriptive data	14*	(a) Give characteristics of study participants (eg demographic, clinical, social) and information on exposures and potential confounders	8, 9
		(b) Indicate number of participants with missing data for each variable of interest	
Outcome data	15*	Report numbers of outcome events or summary measures	10
Main results	16	(*a*) Give unadjusted estimates and, if applicable, confounder-adjusted estimates and their precision (eg, 95% confidence interval). Make clear which confounders were adjusted for and why they were included	10, 11

For peer review only - http://bmjopen.bmj.com/site/about/guidelines.xhtml

		(*b*) Report category boundaries when continuous variables were categorized	
		(*c*) If relevant, consider translating estimates of relative risk into absolute risk for a meaningful time period	
Other analyses	17	Report other analyses done—eg analyses of subgroups and interactions, and sensitivity analyses	
**Discussion**			
Key results	18	Summarise key results with reference to study objectives	11
Limitations	19	Discuss limitations of the study, taking into account sources of potential bias or imprecision. Discuss both direction and magnitude of any potential bias	2
Interpretation	20	Give a cautious overall interpretation of results considering objectives, limitations, multiplicity of analyses, results from similar studies, and other relevant evidence	
Generalisability	21	Discuss the generalisability (external validity) of the study results	12
**Other information**			
Funding	22	Give the source of funding and the role of the funders for the present study and, if applicable, for the original study on which the present article is based	15

*Give information separately for exposed and unexposed groups.

Note: An Explanation and Elaboration article discusses each checklist item and gives methodological background and published examples of transparent reporting. The STROBE checklist is best used in conjunction with this article (freely available on the Web sites of PLoS Medicine at http://www.plosmedicine.org/, Annals of Internal Medicine at http://www.annals.org/, and Epidemiology at http://www.epidem.com/). Information on the STROBE Initiative is available at www.strobe-statement.org.

For peer review only

BMJ Open

Co-utilization of oral rehydration solution and zinc for treating diarrhea, and its associated factor among under-five children in East Africa: a multilevel robust Poisson regression

Journal:	*BMJ Open*
Manuscript ID	bmjopen-2023-079618.R1
Article Type:	Original research
Date Submitted by the Author:	12-Feb-2024
Complete List of Authors:	Legesse, Bruck Tesfaye; Wollega University, Department of Pediatrics and Neonatal Nursing<br>Wondie, Wubet ; Ambo University College of Medicine and Public Health, Department of Pediatrics and Child Health Nursing; Ambo University, Department of Pediatrics and Child Health Nursing<br>Gedefaw, Gezahagn ; University of Gondar College of Medicine and Health Sciences, Department of Neonatal Health Nursing<br>Workineh, Yakob Tadese; Wolaita Sodo University, Department of Pediatrics and Child Health Nursing<br>Seifu, Beminate; Samara University, Public health
**Primary Subject Heading**:	Public health
Secondary Subject Heading:	Infectious diseases, Paediatrics, Public health
Keywords:	PREVENTIVE MEDICINE, Community child health < PAEDIATRICS, Paediatric infectious disease & immunisation < PAEDIATRICS

SCHOLARONE™
Manuscripts

I, the Submitting Author has the right to grant and does grant on behalf of all authors of the Work (as defined in the below author licence), an exclusive licence and/or a non-exclusive licence for contributions from authors who are: i) UK Crown employees; ii) where BMJ has agreed a CC-BY licence shall apply, and/or iii) in accordance with the terms applicable for US Federal Government officers or employees acting as part of their official duties; on a worldwide, perpetual, irrevocable, royalty-free basis to BMJ Publishing Group Ltd ("BMJ") its licensees and where the relevant Journal is co-owned by BMJ to the co-owners of the Journal, to publish the Work in this journal and any other BMJ products and to exploit all rights, as set out in our licence.

The Submitting Author accepts and understands that any supply made under these terms is made by BMJ to the Submitting Author unless you are acting as an employee on behalf of your employer or a postgraduate student of an affiliated institution which is paying any applicable article publishing charge ("APC") for Open Access articles. Where the Submitting Author wishes to make the Work available on an Open Access basis (and intends to pay the relevant APC), the terms of reuse of such Open Access shall be governed by a Creative Commons licence – details of these licences and which Creative Commons licence will apply to this Work are set out in our licence referred to above.

Other than as permitted in any relevant BMJ Author's Self Archiving Policies, I confirm this Work has not been accepted for publication elsewhere, is not being considered for publication elsewhere and does not duplicate material already published. I confirm all authors consent to publication of this Work and authorise the granting of this licence.

For peer review only

Co-utilization of oral rehydration solution and zinc for treating diarrhea, and its associated factor among under-five children in East Africa: a multilevel robust Poisson regression

Bruck Tesfaye Legesse[1]*, Wubet Tazeb Wondie[2] Gezahagn Demsu Gedefaw[3], Yakob Tadese Workineh[4], Beminate Lemma Seifu[5],

Affiliations

[1] Department of Pediatrics and Neonatal Nursing, School of Nursing and Midwifery, Institute of Health Sciences, Wollega University, Nekemte, Ethiopia.

[2]Department of Pediatrics and Child Health Nursing, College of Medicine and Health Sciences, Ambo University, Ambo, Ethiopia

[3]Department of Neonatal Health Nursing, School of Nursing, College of Medicine and Health Sciences, and Specialized Hospital, University of Gondar, Gondar, Northwest Ethiopia

[4]Department of Pediatrics and Child Health Nursing, School of Nursing, College of Medicine and Health Sciences, Wolaita Sodo University, Wolaita Sodo, Ethiopia.

[5]Department of Public Health, College of Medicine and Health Sciences, Samara University, Samara, Ethiopia.

***Corresponding author:** Bruck Tesfaye Legesse;

Email: **brucktesfaye143@gmail.com**

For peer review only

Abstract

Objective: This study aimed to assess the co-utilization of oral rehydration solution and zinc for treating diarrhoea and its associated factors among under-five children in East Africa.

Design and setting: Recent Demographic and Health Survey (DHS) data from 2011 to 2022 of 12 East African countries were used and a total of 16,850 participants were included in the study. A generalized linear mixed model with robust error variance was used to account for the clustering effects of the DHS data and the binary nature of the outcome variable. An adjusted prevalence ratio with a 95% confidence interval (CI) was reported to declare the statistical significance.

Result: In East African nations, the co-utilization of ORS and zinc for the treatment of diarrhoea in children under five was 53.27% with a 95% CI (52.54%, 54.01%). Children of mothers with primary education (aPR = 1.15, 95% CI = 1.09, 1.20), secondary education (aPR=1.08, 95% CI= 1.02-1.14), higer education (aPR=1.19, 95% CI= 1.10-1.29), those from maternal age category of 20-24 (aPR=1.14, 95% CI= 1.07-1.21), age category of 25-29 (aPR=1.13, 95% CI= 1.06-1.21), age category of 30-34 (aPR=1.09, 95% CI= 1.02-1.16), those from wealthy households (aPR = 1.04, 95% CI = 1.01, 1.09), and those who have a media exposure (aPR=1.04, 95% CI= 1.01-1.08) were more likely to receive combination.

Conclusion: Only half of the under-five children with diarrhoea in East Africa were treated with a combination of ORS and zinc. To increase the use of the suggested combination therapy of ORS with zinc, it is important to empower women through education and prevent teen pregnancy.

Keywords: Associated factors; Diarrhoea; East Africa: multilevel analysis; ORS and Zinc co-utilization; under-five children

For peer review only

Strength and limitation

- This study was based on nationally representative data and used appropriate statistical analysis techniques.
- This is also the first study to look at the factors that influence ORS and zinc co-utilization in children under the age of five in East Africa.
- Despite the aforementioned strengths, the measure of zinc utilization practice was based on the mother's recall, which could lead to recall bias.
- Second, the DHS survey lacks information on zinc availability and the role of diet in zinc intake. As a result, we are unable to evaluate these factors.
- Some East African countries are not included in the DHS program. Furthermore, due to the cross-sectional nature of the data, a clear temporality (cause and effect relationship) between the dependent and independent variables was not established.

Background

Diarrhea is three or more loose or watery stools in a 24-hour period, which can also present as a change in the volume, frequency, or fluidity of faeces (1). Although there are easy ways to prevent and cure diarrhoea in children, it continues to be the world's greatest cause of mortality and under nutrition in children under the age of five (2). Each year, around 525,000 children under the age of five lose their lives to diarrhoea (3), and 60% of these deaths occur in ten countries in Asia and Africa: Bangladesh, Democratic Republic of the Congo, Ethiopia, India, Kenya, Niger, Nigeria, Pakistan, Tanzania, and Uganda. Low and middle-income countries (LMICs) have a high fatality rate from diarrhoea because the condition is common there and is linked to high-risk factors such malnutrition, contaminated water sources, and a lack of access to necessary care (2).

For peer review only

One of the top priorities for reducing unnecessary child deaths under the Sustainable Development Goal is treating diarrhoea (4). Up to 93% of diarrhoea-related death can be avoided using ORS and zinc, two very affordable and extremely effective diarrhoea therapies. Because of this, the World Health Organisation (WHO) and the United Nations Children's Fund (UNICEF) suggest combining ORS with zinc for a successful therapy (5, 6). Co-packaging zinc with ORS has the potential to promote their combined usage, improve treatment access, and maximize treatment use (7). The combination of zinc and ORS in a visually pleasing co-pack with counselling messages and instructions greatly enhanced healthcare providers' prescribing practices and parents' compliance with zinc administration (8). Another study conducted in Cambodia reached the same conclusion: co-packaging is a useful strategy for promoting the combined utilization of ORS and zinc (9).

Zinc supplementation lessens the length and intensity of episodes and lowers the likelihood of recurrence in the short term (10, 11), ORS contains trisodium citrate dehydrate, glucose, sodium chloride, and potassium chloride which is important to replenishes the vital fluids and salts lost via diarrhoea (12). A study conducted on zinc supplementation at a Dhaka hospital (International Centre for Diarrhoeal Disease Research, Bangladesh) indicated that dehydration, bloody diarrhoea and fever were less with those under five children received a zinc for diarrhoea at home (13).

However, the global prevalence of using ORS with zinc to treat diarrhoea continues to be alarmingly low (2). Less than 7% of children under the age of five in LMICs receive zinc treatment, according to a 2017 UNICEF report, and only 44% of them receive ORS (14). Access to this life-saving medicine might be made easier in many of these nations, many of which have high mortality rates from diarrhoea (15).

For peer review only

In East Africa, only a small percentage of under-five children receive effective treatment or ORS with Zinc for diarrheal disease. A study in Kenya mentioned that children treated with bundled ORS with zinc accounts for 15% (16), while another study in Ethiopia found only 16.65% (17). Another study conducted in Uganda mentioned that 30% of those under five children treated for diarrhea took co-packed ORS with zinc (18).

According to studies done so far maternal education, economic status, media exposure, residency, proximity to a medical facility, and health insurance coverage have all been found to influence the combination of Zinc and ORS for the treatment of diarrhea in under five children (17, 19-21).

Despite the clinical significance of using ORS and zinc together, and the recommendation of WHO and UNICEF for co-utilization of ORS and zinc, there is limited evidence of no research that ascertains the prevalence and contributing variables of ORS and zinc co-use among young children with diarrhoea in East Africa. So, in order to bridge this gap, a study was conducted. Since this study is the first of its kind in East Africa, policymakers and those making decisions may potentially use it as a starting point when deciding how best to encourage uptake.

Methods

Data source, study setting, and population

This study was based on the most recent Demographic and Health Survey (DHS) data from 2011 to 2022 of 12 East African countries. Those countries are Burundi, Ethiopia, Kenya, Comoros, Madagascar, Malawi, Tanzania, Uganda, Zambia, and Zimbabwe. DHS is a nationally representative survey routinely conducted every five years and collects data on basic health indicators like mortality, morbidity, fertility, and maternal and child health-related characteristics. The study participants were selected using a two-stage stratified sampling technique used for the

For peer review only

survey. In the first stage, Enumeration Areas (EAs) were randomly selected based on the country's recent population, and using the housing census as a sampling frame, households were randomly selected in the second stage. Different datasets, including those for men, women, children, births, and households, are included in each nation's survey. For this study, the study population was under five children, thus, we used the kid's record dataset (KR file). All children under five children in East Africa who had diarrhea was the study's source population. All under-five children who had diarrhea in the two weeks before the survey were the study population. In the current study, a weighted sample of 16,850 under-five children was considered for final analysis. Details about DHS methodology can be accessed at https://dhsprogram.com/Methodology/index.cfm.

Study variables and definitions

Dependent variable: The co-utilization of ORS and zinc was dichotomized as "yes=1" if the child uses both ORS and zinc for the treatment of childhood diarrhea and "no=0" if the child does not use both ORS and zinc for the treatment of childhood diarrhea.

Independent variables: The independent variables were classified as community and individual-level variables. Individual-level factors were maternal age, educational status of the mother, father's educational status, working status of the mother, wealth status, media exposure, sex of household head, health insurance, and sex of the child. Distance to health facility, place of residence (urban or rural), community women's education, and community poverty level were considered as community-level factors.

Operational definition

Media exposure: was created from three variables (frequency of listening to the radio, watching television, and reading newspapers or magazines). In this study, women who listened to radio

For peer review only

watched television, or read newspapers/magazines at least less than once a week were considered as having exposure to media (coded "Yes") and otherwise labeled as not having media exposure (coded "No").

Community women's education: defined as the proportion of women in the cluster who pursued education at the primary, secondary, and higher education levels. The aggregate of individual women's primary, secondary, and higher educational attainment can show the overall educational status of women within the cluster. They were categorized into two categories a higher proportion of women's education within the cluster and a lower proportion of women's education based on the national median value.

Community poverty status: defined as the proportion of poor and poorest mothers within the cluster also can be defined as the household wealth index for each child. For each cluster proportion of poor and poorest was aggregated and show overall poverty status within the cluster. It was categorized into two categories based on the national median value a higher proportion of poor/poorest mothers and a lower proportion of mothers within a cluster.

Data management and statistical analysis

Data extraction, coding, and analysis were done using Stata version 17 statistical software. To restore the data's representativeness, an analysis was conducted using the weighted data. Considering the hierarchical structure of the DHS data, the clustering effect was assessed by estimating the Intra-class Correlation Coefficient (ICC). A significant clustering effect was observed according to the ICC (ICC>10%). This study was a cross-sectional study and the prevalence of co-utilization of ORS and zinc was greater than 10%, and if we report the odds ratio, it could overestimate the association between co-utilization of ORS and zinc and the independent

For peer review only

variables. As the prevalence ratio is the best measure of association in these situations, multilevel Poisson regression analysis with robust variance was fitted to identify predictors of zinc and ORS co-utilization among under-five children with diarrhoea. In the bi-variable multilevel Poisson regression analysis, variables having a p-value<0.2 were considered for the multivariable analysis.

For the multilevel Poisson regression analysis, four models were constructed. The first model aimed to ascertain the degree of cluster variation in the co-utilization of zinc and ORS and it was a null model devoid of explanatory factors. The second model was fitted with individual-level variables, the third with community-level variables, and the fourth with both individual and community-level variables at the same time. Because the models were nested, the deviance -2Log-Likelihood Ratio (LLR) was used to compare them, and the model with the lowest deviance was the best-fitted model for the data. Finally, the 95% confidence interval (CI) for the adjusted prevalence ratio (APR) was determined, and factors in the multivariable analysis with a p-value of < 0.05 were deemed significant predictors of the co-utilization of zinc and ORS among under-five children with diarrhoea.

Patient and public involvement

In this study, the study participants and/or the public were not directly involved in the design, conduct, reporting, and dissemination of this work.

Results

Socio-demographic characteristics

A total weighted sample of 16,850 under-five children who had diarrhea within 2 weeks preceding the survey were included in this study. More than half, 8,965 (53.20%) of the children were males

For peer review only

while the vast majority (79.10%) of them resided in rural areas. The majority, 7,778 (46.16%) were from poor households and 3,666 (21.76%) had mothers who did not have a formal education. Only 818 (5.72%) were covered by health insurance (*Table 1*).

Table 1: Socio-demographic and economic characteristics of study participants in East Africa from 2011 to 2022.

Variable	Weighted frequency	Percent
**Individual level variables**		
**Sex of the child**		
**Male child**	8,965	53.20
**Female child**	7,885	46.80
**Age of the mother**		
**15-19**	1,433	8.51
**20-24**	4,715	27.98
**25-29**	4,545	26.97
**30-34**	3,228	19.16
**35-39**	1,965	11.66
**40-44**	757	4.49
**45-49**	206	1.22
**Marital status**		
**Never in union**	965	5.73
**Married/ living together**	14,160	84.03
**Divorced/widowed/ separated**	1,725	10.24

For peer review only

**Maternal education**		
**No formal education**	3,666	21.76
**Primary**	8,544	50.71
**Secondary**	3,942	23.40
**Higher**	697	4.14
**Father's education (n=14,164)**		
**No formal education**	2,667	18.83
**Primary**	6,842	48.31
**Secondary**	3,657	25.82
**Higher**	997	7.04
**Maternal current working status**		
**Not Employed**	5,747	34.11
**Employed**	11,103	65.89
**Sex of household head**		
**Male household head**	12,778	75.84
**Female household head**	4,072	24.16
**Household wealth status**		
**Poor**	7,778	46.16
**Middle**	3,218	19.10
**Rich**	5,854	34.74
**Covered by health insurance (n=14,291)**		
**No**	13,472	94.28

For peer review only

**Yes**	818	5.72
**Media exposure**		
**No**	5,959	35.36
**Yes**	10,891	64.64
**Community level variables**		
**Distance to health facility (n=15,498)**		
**Big problem**	6,926	44.69
**Not a big problem**	8,572	55.31
**Residence**		
**Urban**	3,521	20.90
**Rural**	13,329	79.10
**Community educational status**		
**Low**	2,996	17.78
**High**	13,854	82.22
**Community poverty level**		
**Low**	7,695	45.67
**High**	9,155	54.33

For peer review only

The prevalence of ORS and Zinc co-utilization for diarrhea treatment

The pooled prevalence of co-utilization of ORS and zinc for the treatment of diarrhea among under-five children in East African countries was 53.27% with a 95% CI (52.54%, 54.01%). In

East African countries, the prevalence of ORS and zinc co-utilization ranges from 24.36% (95% CI: 21.85, 26.86) in Madagascar to 69.32% (95% CI: 67.76, 70.87) in Malawi (Figure 1).

Figure 1: Forest plot of pooled prevalence of co-utilization of ORS and zinc for management of diarrhoea among under-five children in East Africa from 2011 to 2022.

Random effect and model comparison

To evaluate the random or clustering effect using the intraclass correlation coefficient (ICC), we used deviance for model comparison. The null model's ICC of 0.145 indicated that unmeasured or unmeasurable factors (random effects) accounted for about 14.5% of the variance in the co-utilization of zinc and ORS. In terms of comparing models, the best-fitted model was the final model, which had a lower deviance (Table 2).

Table 2: Random effect and model comparison for factors associated with co-utilization of ORS and zinc for management of diarrhoea among under-five children in East Africa from 2011 to 2022.

	**Null model**	**Model 1**	**Model 2**	**Model 3**
**ICC**	14.56%	15.31%	15.01%	15.01%
**Log-likelihood**	-14402.21	-14369.22	-14398.24	-14367.59
**Deviance**	28,804.42	28,738.44	28,796.48	28,735.18

ICC, intraclass correlation coefficient

Factors associated with co-utilization of ORS and Zinc for under-five diarrhea treatment

A multilevel bi-variable analysis was done to assess the crude relationship between the independent variables and ORS-zinc co-utilization. Among the variables included in the bi-variable analysis only maternal age, maternal education status, wealth index, maternal employment

For peer review only

status, media exposure, community-level illiteracy, and community-level poverty showed a statistically significant association with ORS and zinc co-utilization at a *p*-value of < 0.20.

In the final model maternal age, maternal education status, wealth index, and media exposure were significantly associated with ORS and zinc co-utilization (p-value ≤ 0.05). The prevalence of ORS and zinc co-utilization was 1.14 (APR=1.14, 95%CI: 1.07, 1.21), 1.13 (APR=1.13, 95%CI: 1.06, 1.21), and 1.09 (APR=1.09, 95%CI: 1.02, 1.16) times higher in children whose mothers were aged 20-24, 25-29, and 30-34, respectively in comparison to mother aged 15-19. Being the child of a woman with a primary, secondary, or higher level of education raises the prevalence of co-utilization by 1.15, 1.08, and 1.19, respectively. Compared to children from poor household wealth index, those children from rich wealth index had a 4 % higher prevalence of ORS and zinc co-utilization. The prevalence of ORS and zinc co-utilization among children whose mothers had media exposure was 1.04 times higher as compared to their counterparts (APR = 1.04, 95% CI :1.01, 1.08) (*Table 3*).

Table 3: Factors associated with co-utilization of ORS and zinc for management of diarrhoea among under-five children in East Africa from 2011 to 2022.

**Variable**	**ORS and Zinc Co-utilization**		**CPR (95%CI)**	**APR (95%CI)**	**P-value**
**Individual level variables**	**No (%)**	**Yes (%)**			
**Age of the mother**					
**15-19**	746 (52.03)	687 (47.97)	1	1	
**20-24**	2,106 (44.67)	2,609 (55.33)	1.13 (1.06, 1.2)	1.14 (1.07, 1.2)*	< 0.01
**25-29**	2,026 (44.57)	2,519 (55.43)	1.12 (1.05, 1.2)	1.13 (1.06,1.2)*	< 0.01

For peer review only

**30-34**	1,573 (48.73)	1,654 (51.27)	1.06 (0.99, 1.1)	1.09 (1.02, 1.2)*	< 0.01
**35-39**	982 (49.99)	982 (50.01)	1.01 (0.94, 1.1)	1.04 (0.97, 1.1)	0.256
**40-44**	400 (52.90)	357 (47.10)	0.95 (0.87, 1.0)	1.00 (0.92, 1.1)	0.929
**45-49**	106 (51.32)	100 (48.68)	0.98 (0.85, 1.1)	1.05 (0.91, 1.2)	0.530
**Maternal educational status**					
**No formal education**	2,004 (54.67)	1,662 (45.33)	1	1	
**Primary**	3,873 (45.33)	4,671 (54.67)	1.17 (1.12, 1.2)	1.15 (1.09, 1.2)*	< 0.01
**Secondary**	1,806 (45.81)	2,136 (54.19)	1.14 (1.08, 1.2)	1.08 (1.02, 1.1)*	< 0.08
**Higher**	256 (36.75)	440 (63.25)	1.28 (1.19, 1.4)	1.19 (1.10, 1.3)*	< 0.01
**Household wealth index**					
**Poor**	3,819 (49.10)	3,958 (50.90)	1	1	
**Middle**	1,511 (46.96)	1,707 (53.04)	1.05 (1.01, 1.1)	1.03 (0.99, 1.1)	0.137
**Rich**	2,609 (44.57)	3,245 (55.43)	1.07 (1.03, 1.1)	1.04 (1.01, 1.1)*	0.042
**Maternal employment status**					
**Not employed**	2,769 (48.19)	2,977 (51.81)	1	1	
**Employed**	5,170 (46.57)	5,933 (53.43)	0.98 (0.95, 1.1)	0.97 (0.94, 1.04)	0.08
**Media exposure**					
**No**	3,022 (50.72)	2,936 (49.28)	1	1	
**Yes**	4,917 (45.15)	5,974 (54.85)	1.08 (1.04, 1.1)	1.04 (1.01, 1.1)*	0.046
**Community illiteracy level**					

**Low**	1,287 (42.96)	1,709 (57.04)	1	1	
**High**	6,653 (48.02)	7,201 (51.98)	0.92 (0.89, 0.9)	0.96 (0.92, 1.03)	0.065
**Community poverty level**					
**Low**	3,641 (47.32)	4,053 (52.68)	1	1	
**High**	4,298 (46.95)	4,857 (53.05)	0.99 (0.97, 1.0)	1.03 (0.99, 1.1)	0.095

*p-value ≤ 0.05, CPR: Crude Prevalence Ratio, APR: Adjusted Prevalence Ratio

Discussion

This study aimed to assess the factors associated with the co-use of ORS and zinc for the treatment of diarrhoea in under-five children across East African countries. In our study, the co-use of ORS and zinc was associated with the mother's age, her educational level, family's financial status, and media exposure.

This study mentioned maternal age above twenty is associated with co-utilization of ORS and zinc and this finding is supported by different study conducted in Nigeria (22). The possible reason for this finding is older mothers were more likely than younger mothers to be knowledgeable about pediatric diarrhoea and how to treat it, younger mothers do not have as much exposure as older mothers about child diarrhoea and its management as maternal age can have impact on their knowledge about and how to treat it (23). This study found that maternal educational status was associated with the usage of ORS with zinc to treat under-five diarrheal disease and this finding is supported by studies conducted in Pakistan (24), India (25), Nigeria (19), Cameron (26), Kenya (27), and Ethiopia (17, 21, 28-30). This may be because educated women are more likely to take sick children to the medical facility and a woman with education is more likely than a mother

For peer review only

without education to take her ill kid for follow-up as well as to take the ordered medication as per recommended. In addition to the above reason, educated mothers have a better awareness regarding the health problems of their kids than a mother without education (31, 32).

Our study mentioned that media exposure was one of the factors associated with diarrheal treatment using ORS with zinc. This finding is inline with studies conducted in India (25), Bangladesh (33), Ghana (34), and Ethiopia (21, 30). This may be due to the media's important role in increasing the mother's or caregiver's understanding and awareness of the best ways to treat diarrhoea in children (33).

As this study finds, a family's wealth status affects the usage of ORS with zinc. This finding is supported by different studies conducted in India (35), and Ethiopia (21). This is due to the fact that low-income households in low and middle-income countries (LMICs), such as those in Africa, particularly in East Africa, cannot afford the expenses of healthcare services, including ORS and zinc. Thus, when children from low-income households get diarrhoea, they might not receive ORS with zinc (36).

Conclusion

In this study, media exposure, rich household wealth status, maternal education, and maternal age over twenty were associated factors for ORS and zinc co-use. To raise the prevalence of ORS and zinc co-utilization for the successful treatment of diarrhoea in children under the age of five, it is advised to increase media exposure, maternal education, and support the household wealth index. Using media as tool to advertise the importance of the co-utilization of ORS and zinc for all community will increase and boost the utilization of the product. Empowering women through education also will raise the co-utilization of ORS and zinc. In addition, policies and intervention

For peer review only

programs that aim to stop unnecessary deaths of children under the age of five should be prioritized by preventing adolescent pregnancy.

Abbreviations: aPR, Adjusted Prevalence Ratio; CI, Confidence Interval; uPR, Unadjusted Prevalence Ratio; DHS, Demographic and Health Survey; ICC, Intraclass Correlation Coefficient; LL: Log-likelihood; UNICEF: United Nations Children's Fund

Competing interests

The authors declare that they have no competing interests.

Authors' contributions

Conceptualization: Bruck Tesfaye Legesse, Beminate Lemma Seifu

Data curation: Bruck Tesfaye Legesse, Beminate Lemma Seifu

Formal analysis: Beminate Lemma Seifu, Bruck Tesfaye Legesse

Investigation: Bruck Tesfaye Legesse, Beminate Lemma Seifu

Methodology: Beminate Lemma Seifu, Bruck Tesfaye Legesse

Software: Bruck Tesfaye Legesse, Beminate Lemma Seifu

Validation: Beminate Lemma Seifu, Bruck Tesfaye Legesse, Wubet Tazeb Wondie, Gezahagn Demsu Gedefaw, Yakob Tadese Workineh

Writing – original draft: Bruck Tesfaye Legesse, Beminate Lemma Seifu

Writing – review & editing: Beminate Lemma Seifu, Bruck Tesfaye Legesse, Wubet Tazeb Wondie, Gezahagn Demsu Gedefaw, Yakob Tadese Workineh

For peer review only

Acknowledgments

We express our gratitude to the MEASURE DHS for providing the study's dataset.

Funding

No fund was received for this work.

Availability of data and materials

All the data used for the study are available through an online request to http://www.dhsprogram.com

Consent for publication

Not applicable.

Supporting information files

Supporting file 1: STROBE Statement—Checklist of items that should be included in reports of cross-sectional studies

Ethical consideration

Through an online request to http://www.dhsprogram.com, permission to access the measure demographic and health survey data used in this study was obtained. Data from the official program database are accessible to the public.

For peer review only

Reference

1. Guarino A, Dupont C, Gorelov AV, Gottrand F, Lee JK, Lin Z, et al. The management of acute diarrhea in children in developed and developing areas: from evidence base to clinical practice. 2012;13(1):17-26.

2. Almasi A, Zangeneh A, Ziapour A, Saeidi S, Teimouri R, Ahmadi T, et al. Investigating Global Spatial Patterns of Diarrhea-Related Mortality in Children Under Five. 2022;10:861629.

3. Mokomane M, Kasvosve I, Melo Ed, Pernica JM, Goldfarb DMJTaiid. The global problem of childhood diarrhoeal diseases: emerging strategies in prevention and management. 2018;5(1):29-43.

4. Lee BX, Kjaerulf F, Turner S, Cohen L, Donnelly PD, Muggah R, et al. Transforming our world: implementing the 2030 agenda through sustainable development goal indicators. 2016;37:13-31.

5. Qazi S, Aboubaker S, MacLean R, Fontaine O, Mantel C, Goodman T, et al. Ending preventable child deaths from pneumonia and diarrhoea by 2025. Development of the integrated Global Action Plan for the Prevention and Control of Pneumonia and Diarrhoea. 2015;100(Suppl 1):S23-S8.

6. Black R, Fontaine O, Lamberti L, Bhan M, Huicho L, El Arifeen S, et al. Drivers of the reduction in childhood diarrhea mortality 1980-2015 and interventions to eliminate preventable diarrhea deaths by 2030. 2019;9(2).

7. Hill S, Yang A, Bero L. Priority medicines for maternal and child health: a global survey of national essential medicines lists. PloS one. 2012;7(5):e38055.

8. Roche M, García Meza R, Vossenaar M. An intervention to Co-package zinc and oral rehydration salts (ORS) improves health provider prescription and maternal adherence to

For peer review only

WHO-recommended diarrhea treatment in Western Guatemala. The FASEB Journal. 2015;29:902.23.

9. Borapich D, Warsh M, Borapich D, Warsh M. Improving child health in Cambodia: social marketing of diarrhea treatment kit, results of a pilot project. Cases in Public Health Communication & Marketing. 2010;4(4):22.

10. Barffour MA, Hinnouho G-M, Wessells KR, Kounnavong S, Ratsavong K, Sitthideth D, et al. Effects of therapeutic zinc supplementation for diarrhea and two preventive zinc supplementation regimens on the incidence and duration of diarrhea and acute respiratory tract infections in rural Laotian children: A randomized controlled trial. Journal of global health. 2020;10(1).

11. Lazzerini M, Wanzira H. Oral zinc for treating diarrhoea in children. Cochrane database of systematic reviews. 2016(12).

12. Organization WH. Ending preventable child deaths from pneumonia and diarrhoea by 2025: the integrated global action plan for pneumonia and diarrhoea (GAPPD). 2013.

13. Alam J, Nuzhat S, Billal SM, Ahmed T, Khan AI, Hossain MI. Nutritional Profiles and Zinc Supplementation among Children with Diarrhea in Bangladesh. The American journal of tropical medicine and hygiene. 2023;108(4):837-43.

14. Gona PN, Gona CM, Chikwasha V, Haruzivishe C, Rao SR, Mapoma CC. Oral rehydration solution coverage in under 5 children with diarrhea: a tri-country, subnational, cross-sectional comparative analysis of two demographic health surveys cycles. BMC Public Health. 2020;20(1):1-15.

For peer review only

15. Mohamed SOO, Alawad MOA, Ahmed AAM, Mahmoud AAAJBRN. Access to oral rehydration solution and zinc supplementation for treatment of childhood diarrhoeal diseases in Sudan. BMC Research Notes. 2020;13.

16. Lam F, Wentworth L, Cherutich P, Migiro S, Abdala K, Musyoka M, et al. An evaluation of a national oral rehydration solution and zinc scale-up program in Kenya between 2011 and 2016. 2019;9(1).

17. Kassa SF, Alemu TG, Techane MA, Wubneh CA, Assimamaw NT, Belay GM, et al. The co-utilization of oral rehydration solution and zinc for treating diarrhea and its associated factors among under-five children in Ethiopia: further analysis of EDHS 2016. 2022:1713-21.

18. Lam F, Kirchhoffer D, Buluma DM, Kabunga L, Wamala-Mucheri PN, Schroder K, et al. A retrospective mixed-methods evaluation of a national ORS and zinc scale-up program in Uganda between 2011 and 2016. 2019;9(1).

19. Gwarzo GJNJoP. Mothers' awareness and use of zinc in under-five children with diarrhoea in North-Western Nigeria. 2018;45(2):81-5.

20. Ajayi DJAoB, Medicine A. Determinants of Oral Rehydration Solution and Zinc Use Among Under-Five Children for The Management of Diarrhea in Abeokuta, Nigeria. 2019;7(1):35–9-–9.

21. Negesse Y, Fetene Abebe G, Addisu A, Setegn Alie M, Alemayehu DJFiPH. The magnitude of oral rehydration salt utilization in diarrhea hot spot regions of Ethiopia and its associated factors among under-five children: A multilevel analysis based on Bayesian approach. 2022;10:960627.

For peer review only

22. Okafor IP, Akinyemi OT, Wika-Kobani BN, Olubodun T, Eze UT. Childhood diarrhoea: a cross-sectional survey on maternal knowledge, hygienic practices and use of oral zinc for home management in a Nigerian community. The Pan African medical journal. 2022;42:123.

23. Okoh BA, Alex-Hart BA. Home management of diarrhoea by caregivers presenting at the diarrhoea training unit of a tertiary hospital in southern Nigeria. British Journal of Medicine and Medical Research. 2014;4(35):5524-40.

24. Duggal MN, Bari A, Mansoor A, Saeed S, Rizwan W, Nayar JJJTJotPMA. Awareness of quantity of ORS and Zinc among mothers of under five children with diarrhoea at a tertiary care Centre Lahore. 2021;71(3):921-4.

25. Lam F, Pro G, Agrawal S, Shastri VD, Wentworth L, Stanley M, et al. Effect of enhanced detailing and mass media on community use of oral rehydration salts and zinc during a scale-up program in Gujarat and Uttar Pradesh. 2019;9(1).

26. Essomba NE, Koum DK, Adiogo D, Ngwe M, Coppieters YJMmj. Use of oral rehydration therapy in the treatment of childhood diarrhoea in Douala, Cameroon. 2015;27(2):60-4.

27. Njeri G, Muriithi MJESJ. Household choice of diarrhea treatments for children under the age of five in Kenya: evidence from The Kenya Demographic and Health Survey 2008-09. 2013;9(6):77-91.

28. Ayele E, Tasew H, Mariye T, Teklay G, Alemayhu T, Mesfin FJM. Zinc utilization and associated factors among under-five children having acute diarrhea in Kebri-dehar Town, Somali Region, Ethiopia-2017. 2020;4(1):15-9.

29. Terefa DR, Shama AT, Kenea AKJBo. Sociodemographic and institutional determinants of zinc bundled with oral rehydration salt utilisation among under-five children with diarrhoeal

For peer review only

diseases in East Wallaga zone, western Ethiopia: a community-based cross-sectional study. 2023;13(5):e070203.

30. Teshale AB, Liyew AM, Tesema GAJBPH. Factors associated with zinc utilization for the management of diarrhea in under-five children in Ethiopia. 2020;20:1-7.

31. Adane M, Mengistie B, Mulat W, Kloos H, Medhin GJJoH, Population, Nutrition. Utilization of health facilities and predictors of health-seeking behavior for under-five children with acute diarrhea in slums of Addis Ababa, Ethiopia: a community-based cross-sectional study. 2017;36(1):1-12.

32. Apuleni G, Jacobs C, Musonda P. Predictors of health seeking behaviours for common childhood illnesses in poor resource settings in Zambia, a community cross sectional study. Frontiers in Public Health. 2021;9:569569.

33. Alam Z, Higuchi M, Sarker MAB, Hamajima NJNjoms. Mass media exposure and childhood diarrhea: a secondary analysis of the 2011 Bangladesh demographic and health survey. 2019;81(1):31.

34. El-Khoury M, Banke K, Sloane PJGHS, Practice. Improved childhood diarrhea treatment practices in Ghana: a pre-post evaluation of a comprehensive private-sector program. 2016;4(2):264-75.

35. Bawankule R, Singh A, Kumar K, Pedgaonkar SJBph. Disposal of children's stools and its association with childhood diarrhea in India. 2017;17:1-9.

36. Braimoh T, Danat I, Abubakar M, Ajeroh O, Stanley M, Wiwa O, et al. Private health care market shaping and changes in inequities in childhood diarrhoea treatment coverage: evidence from the analysis of baseline and endline surveys of an ORS and zinc scale-up program in Nigeria. 2021;20:1-12.

For peer review only

For peer review only

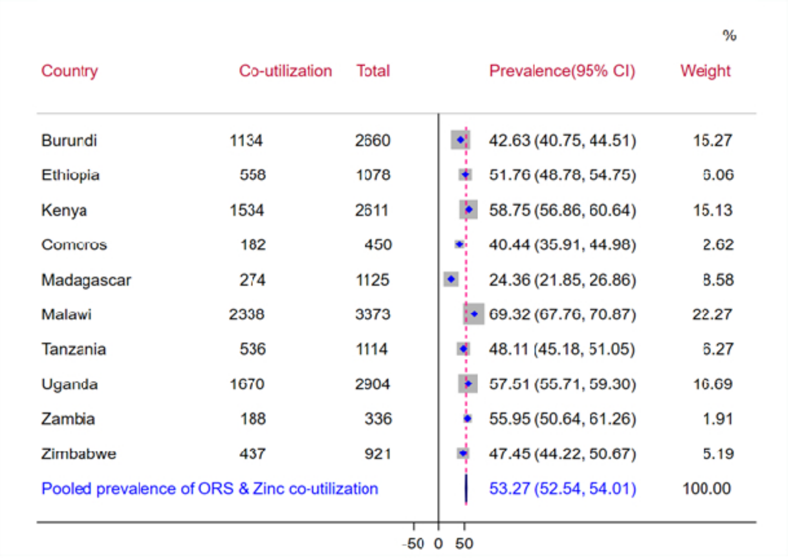

204x120mm (96 x 96 DPI)

STROBE Statement—Checklist of items that should be included in reports of ***cross-sectional studies***

	Item No	Recommendation	Page No
**Title and abstract**	1	(*a*) Indicate the study's design with a commonly used term in the title or the abstract	1
		(*b*) Provide in the abstract an informative and balanced summary of what was done and what was found	2
**Introduction**			
Background/rationale	2	Explain the scientific background and rationale for the investigation being reported	2
Objectives	3	State specific objectives, including any prespecified hypotheses	1, 5
**Methods**			
Study design	4	Present key elements of study design early in the paper	5, 6
Setting	5	Describe the setting, locations, and relevant dates, including periods of recruitment, exposure, follow-up, and data collection	5
Participants	6	(*a*) Give the eligibility criteria, and the sources and methods of selection of participants	5
Variables	7	Clearly define all outcomes, exposures, predictors, potential confounders, and effect modifiers. Give diagnostic criteria, if applicable	6, 7
Data sources/ measurement	8*	For each variable of interest, give sources of data and details of methods of assessment (measurement). Describe comparability of assessment methods if there is more than one group	*7*
Bias	9	Describe any efforts to address potential sources of bias	7
Study size	10	Explain how the study size was arrived at	6, 7
Quantitative variables	11	Explain how quantitative variables were handled in the analyses. If applicable, describe which groupings were chosen and why	
Statistical methods	12	(*a*) Describe all statistical methods, including those used to control for confounding	7
		(*b*) Describe any methods used to examine subgroups and interactions	
		(*c*) Explain how missing data were addressed	
		(*d*) If applicable, describe analytical methods taking account of sampling strategy	
		(*e*) Describe any sensitivity analyses	
**Results**			
Participants	13*	(a) Report numbers of individuals at each stage of study—eg numbers potentially eligible, examined for eligibility, confirmed eligible, included in the study, completing follow-up, and analysed	5
		(b) Give reasons for non-participation at each stage	
		(c) Consider use of a flow diagram	
Descriptive data	14*	(a) Give characteristics of study participants (eg demographic, clinical, social) and information on exposures and potential confounders	8, 9
		(b) Indicate number of participants with missing data for each variable of interest	
Outcome data	15*	Report numbers of outcome events or summary measures	10
Main results	16	(*a*) Give unadjusted estimates and, if applicable, confounder-adjusted estimates and their precision (eg, 95% confidence interval). Make clear which confounders were adjusted for and why they were included	10, 11

For peer review only

		(*b*) Report category boundaries when continuous variables were categorized	
		(*c*) If relevant, consider translating estimates of relative risk into absolute risk for a meaningful time period	
Other analyses	17	Report other analyses done—eg analyses of subgroups and interactions, and sensitivity analyses	
**Discussion**			
Key results	18	Summarise key results with reference to study objectives	11
Limitations	19	Discuss limitations of the study, taking into account sources of potential bias or imprecision. Discuss both direction and magnitude of any potential bias	2
Interpretation	20	Give a cautious overall interpretation of results considering objectives, limitations, multiplicity of analyses, results from similar studies, and other relevant evidence	
Generalisability	21	Discuss the generalisability (external validity) of the study results	12
**Other information**			
Funding	22	Give the source of funding and the role of the funders for the present study and, if applicable, for the original study on which the present article is based	15

*Give information separately for exposed and unexposed groups.

Note: An Explanation and Elaboration article discusses each checklist item and gives methodological background and published examples of transparent reporting. The STROBE checklist is best used in conjunction with this article (freely available on the Web sites of PLoS Medicine at http://www.plosmedicine.org/, Annals of Internal Medicine at http://www.annals.org/, and Epidemiology at http://www.epidem.com/). Information on the STROBE Initiative is available at www.strobe-statement.org.

For peer review only

Co-utilization of oral rehydration solution and zinc for treating diarrhea, and its associated factor among under-five children in East Africa: a multilevel robust Poisson regression

Journal:	*BMJ Open*
Manuscript ID	bmjopen-2023-079618.R2
Article Type:	Original research
Date Submitted by the Author:	19-Feb-2024

Complete List of Authors:	Legesse, Bruck Tesfaye; Wollega University, Department of Pediatrics and Neonatal Nursing<br>Wondie, Wubet ; Ambo University College of Medicine and Public Health, Department of Pediatrics and Child Health Nursing; Ambo University, Department of Pediatrics and Child Health Nursing<br>Gedefaw, Gezahagn ; University of Gondar College of Medicine and Health Sciences, Department of Neonatal Health Nursing<br>Workineh, Yakob Tadese; Wolaita Sodo University, Department of Pediatrics and Child Health Nursing<br>Seifu, Beminate; Samara University, Public health

**Primary Subject Heading**:	Public health
Secondary Subject Heading:	Infectious diseases, Paediatrics, Public health
Keywords:	PREVENTIVE MEDICINE, Community child health < PAEDIATRICS, Paediatric infectious disease & immunisation < PAEDIATRICS

SCHOLARONE™ Manuscripts

I, the Submitting Author has the right to grant and does grant on behalf of all authors of the Work (as defined in the below author licence), an exclusive licence and/or a non-exclusive licence for contributions from authors who are: i) UK Crown employees; ii) where BMJ has agreed a CC-BY licence shall apply, and/or iii) in accordance with the terms applicable for US Federal Government officers or employees acting as part of their official duties; on a worldwide, perpetual, irrevocable, royalty-free basis to BMJ Publishing Group Ltd ("BMJ") its licensees and where the relevant Journal is co-owned by BMJ to the co-owners of the Journal, to publish the Work in this journal and any other BMJ products and to exploit all rights, as set out in our licence.

The Submitting Author accepts and understands that any supply made under these terms is made by BMJ to the Submitting Author unless you are acting as an employee on behalf of your employer or a postgraduate student of an affiliated institution which is paying any applicable article publishing charge ("APC") for Open Access articles. Where the Submitting Author wishes to make the Work available on an Open Access basis (and intends to pay the relevant APC), the terms of reuse of such Open Access shall be governed by a Creative Commons licence – details of these licences and which Creative Commons licence will apply to this Work are set out in our licence referred to above.

Other than as permitted in any relevant BMJ Author's Self Archiving Policies, I confirm this Work has not been accepted for publication elsewhere, is not being considered for publication elsewhere and does not duplicate material already published. I confirm all authors consent to publication of this Work and authorise the granting of this licence.

For peer review only

Co-utilization of oral rehydration solution and zinc for treating diarrhea, and its associated factor among under-five children in East Africa: a multilevel robust Poisson regression

Bruck Tesfaye Legesse[1]*, Wubet Tazeb Wondie[2] Gezahagn Demsu Gedefaw[3], Yakob Tadese Workineh[4], Beminate Lemma Seifu[5],

Affiliations

[1] Department of Pediatrics and Neonatal Nursing, School of Nursing and Midwifery, Institute of Health Sciences, Wollega University, Nekemte, Ethiopia.

[2]Department of Pediatrics and Child Health Nursing, College of Medicine and Health Sciences, Ambo University, Ambo, Ethiopia

[3]Department of Neonatal Health Nursing, School of Nursing, College of Medicine and Health Sciences, and Specialized Hospital, University of Gondar, Gondar, Northwest Ethiopia

[4]Department of Pediatrics and Child Health Nursing, School of Nursing, College of Medicine and Health Sciences, Wolaita Sodo University, Wolaita Sodo, Ethiopia.

[5]Department of Public Health, College of Medicine and Health Sciences, Samara University, Samara, Ethiopia.

***Corresponding author:** Bruck Tesfaye Legesse;

Email: **brucktesfaye143@gmail.com**

For peer review only

Abstract

Objective: This study aimed to assess the co-utilization of oral rehydration solution and zinc for treating diarrhoea and its associated factors among under-five children in East Africa.

Design: Cross-sectional study design. Multilevel Poisson regression analysis with robust variance was fitted to identify predictors of zinc and ORS co-utilization. An adjusted prevalence ratio with a 95% confidence interval (CI) was reported to declare the statistical significance.

Setting: Twelve East African countries.

Participants: 16,850 under-five children who had diarrhea were included in the study.

Result: In East African nations, the co-utilization of ORS and zinc for the treatment of diarrhoea in children under five was 53.27% with a 95% CI (52.54%, 54.01%). Children of mothers with primary education (aPR = 1.15, 95% CI = 1.09, 1.20), secondary education (aPR=1.08, 95% CI= 1.02-1.14), higer education (aPR=1.19, 95% CI= 1.10-1.29), those from maternal age category of 20-24 (aPR=1.14, 95% CI= 1.07-1.21), age category of 25-29 (aPR=1.13, 95% CI= 1.06-1.21), age category of 30-34 (aPR=1.09, 95% CI= 1.02-1.16), those from wealthy households (aPR = 1.04, 95% CI = 1.01, 1.09), and those who have a media exposure (aPR=1.04, 95% CI= 1.01-1.08) were more likely to receive combination.

Conclusion: Only half of the under-five children with diarrhoea in East Africa were treated with a combination of ORS and zinc. To increase the use of the suggested combination therapy of ORS with zinc, it is important to empower women through education and prevent teen pregnancy.

Keywords: Associated factors; Diarrhoea; East Africa: multilevel analysis; ORS and Zinc co-utilization; under-five children

For peer review only

Strength and limitation

- This study was based on nationally representative large data and used appropriate statistical analysis techniques.
- Despite the aforementioned strengths, the measure of zinc utilization practice was based on the mother's recall, which could lead to recall bias.
- Second, the DHS survey lacks information on zinc availability and the role of diet in zinc intake. As a result, we are unable to evaluate these factors.
- Some East African countries are not included in the DHS program. Furthermore, due to the cross-sectional nature of the data, a clear temporality (cause and effect relationship) between the dependent and independent variables was not established.

Background

Diarrhea is three or more loose or watery stools in a 24-hour period, which can also present as a change in the volume, frequency, or fluidity of faeces (1). Although there are easy ways to prevent and cure diarrhoea in children, it continues to be the world's greatest cause of mortality and undernutrition in children under the age of five (2). Each year, around 525,000 children under the age of five lose their lives to diarrhoea (3), and the data taken from the public World Health Organization (WHO) dataset, which included information from 195 nations between 2000 and 2017 findings showed that the largest frequency of diarrhea-related mortality was found in Asian and African nations. The results of the spatial modelling also showed that, up until 2010, the majority of diarrhea-related deaths occurred in Asian nations; however, in 2011, the majority of deaths moved to Africa (2). (2). In East Africa diarrheal-related under-five mortality accounts from

For peer review only

2.1% in Tanzania (4), 8% in Mozambique (5), 8.8% in Ethiopia (6), 9% in Kenya (7), and 18.2 % in Uganda (4).

One of the top priorities for reducing unnecessary child deaths under the Sustainable Development Goal is treating diarrhoea (8). Up to 93% of diarrhoea-related death can be avoided using ORS and zinc, two very affordable and extremely effective diarrhoea therapies. Because of this, the World Health Organisation (WHO) and the United Nations Children's Fund (UNICEF) suggest combining ORS with zinc for a successful therapy (9, 10). Co-packaging zinc with ORS has the potential to promote their combined usage, improve treatment access, and maximize treatment use (11). The combination of zinc and ORS in a visually pleasing co-pack with counselling messages and instructions greatly enhanced healthcare providers' prescribing practices and parents' compliance with zinc administration (12). Another study conducted in Cambodia reached the same conclusion: co-packaging is a useful strategy for promoting the combined utilization of ORS and zinc (13).

Zinc supplementation lessens the length and intensity of episodes and lowers the likelihood of recurrence in the short term (14, 15), ORS contains trisodium citrate dehydrate, glucose, sodium chloride, and potassium chloride which is important to replenishes the vital fluids and salts lost via diarrhoea (16). A study conducted on zinc supplementation at a Dhaka hospital (International Centre for Diarrhoeal Disease Research, Bangladesh) indicated that dehydration, bloody diarrhoea and fever were less with those under five children received a zinc for diarrhoea at home (17).

However, the global prevalence of using ORS with zinc to treat diarrhoea continues to be alarmingly low (2). Less than 7% of children under the age of five in LMICs receive zinc treatment, according to a 2017 UNICEF report, and only 44% of them receive ORS (18). Access to this life-

For peer review only

saving medicine might be made easier in many of these nations, many of which have high mortality rates from diarrhoea (19).

In East Africa, only a small percentage of under-five children receive effective treatment or ORS with Zinc for diarrheal disease. A study in Kenya mentioned that children treated with bundled ORS with zinc accounts for 15% (20), while another study in Ethiopia found only 16.65% (21). Another study conducted in Uganda mentioned that 30% of those under five children treated for diarrhea took co-packed ORS with zinc (22).

According to studies done so far maternal education, economic status, media exposure, residency, proximity to a medical facility, and health insurance coverage have all been found to influence the combination of Zinc and ORS for the treatment of diarrhea in under five children (21, 23-25).

Despite the clinical significance of using ORS and zinc together, and the recommendation of WHO and UNICEF for co-utilization of ORS and zinc, there is limited evidence of no research that ascertains the prevalence and contributing variables of ORS and zinc co-use among young children with diarrhoea in East Africa. So, in order to bridge this gap, a study was conducted. Since this study is the first of its kind in East Africa, policymakers and those making decisions may potentially use it as a starting point when deciding how best to encourage uptake.

Methods

Data source, study setting, and population

This study was based on the most recent Demographic and Health Survey (DHS) data from 2011 to 2022 of 12 East African countries. Those countries are Burundi, Ethiopia, Kenya, Comoros, Madagascar, Malawi, Tanzania, Uganda, Zambia, and Zimbabwe. DHS is a nationally representative survey routinely conducted every five years and collects data on basic health

For peer review only

indicators like mortality, morbidity, fertility, and maternal and child health-related characteristics. The study participants were selected using a two-stage stratified sampling technique used for the survey. In the first stage, Enumeration Areas (EAs) were randomly selected based on the country's recent population, and using the housing census as a sampling frame, households were randomly selected in the second stage. Different datasets, including those for men, women, children, births, and households, are included in each nation's survey. For this study, the study population was under five children, thus, we used the kid's record dataset (KR file). All children under five children in East Africa who had diarrhea was the study's source population. All under-five children who had diarrhea in the two weeks before the survey were the study population. In the current study, a weighted sample of 16,850 under-five children was considered for final analysis. Details about DHS methodology can be accessed at https://dhsprogram.com/Methodology/index.cfm.

Study variables and definitions

Dependent variable: The co-utilization of ORS and zinc was dichotomized as "yes=1" if the child uses both ORS and zinc for the treatment of childhood diarrhea and "no=0" if the child does not use both ORS and zinc for the treatment of childhood diarrhea.

Independent variables: The independent variables were classified as community and individual-level variables. Individual-level factors were maternal age, educational status of the mother, father's educational status, working status of the mother, wealth status, media exposure, sex of household head, health insurance, and sex of the child. Distance to health facility, place of residence (urban or rural), community women's education, and community poverty level were considered as community-level factors.

For peer review only

Operational definition

Media exposure: was created from three variables (frequency of listening to the radio, watching television, and reading newspapers or magazines). In this study, women who listened to radio watched television, or read newspapers/magazines at least less than once a week were considered as having exposure to media (coded "Yes") and otherwise labeled as not having media exposure (coded "No").

Community women's education: defined as the proportion of women in the cluster who pursued education at the primary, secondary, and higher education levels. The aggregate of individual women's primary, secondary, and higher educational attainment can show the overall educational status of women within the cluster. They were categorized into two categories a higher proportion of women's education within the cluster and a lower proportion of women's education based on the national median value.

Community poverty status: defined as the proportion of poor and poorest mothers within the cluster also can be defined as the household wealth index for each child. For each cluster proportion of poor and poorest was aggregated and show overall poverty status within the cluster. It was categorized into two categories based on the national median value a higher proportion of poor/poorest mothers and a lower proportion of mothers within a cluster.

Data management and statistical analysis

Data extraction, coding, and analysis were done using Stata version 17 statistical software. To restore the data's representativeness, an analysis was conducted using the weighted data. Considering the hierarchical structure of the DHS data, the clustering effect was assessed by estimating the Intra-class Correlation Coefficient (ICC). ICC helps to determine whether there is

For peer review only

a clustering effect present in the DHS data, which has a hierarchical structure. It is important to account for any clustering effect in the model to ensure accurate analysis. A significant clustering effect was observed according to the ICC (ICC>10%). Multilevel models are a statistical technique that is used to analyze data with hierarchical or clustered structures. Traditional multiple regression techniques treat observations as independent, which can lead to inaccurate results. In contrast, multilevel models account for the hierarchy of the data, which results in more accurate standard errors for regression coefficients. This makes them a powerful tool for analyzing data with complex structures. In statistical analysis, the act of ignoring clustering effects can lead to an overstatement of statistical significance. Our study has demonstrated that when studying the co-utilization of individuals, multilevel models can provide a reliable approach to identifying cluster-level effects based on country or enumeration area. By leveraging such models, we can better understand the nature of clustering and its impact on statistical significance, thereby ensuring the validity of our results. When the outcome is prevalent in the population, such as in the case where more than 10% of the population has co-utilization of ORS and Zinc, the prevalence ratio (PR) is considered the preferred method of measuring the association between exposure and outcome. The PR provides a direct measure of how much more or less likely individuals with the exposure are to have the condition compared to those without the exposure. In our study, the odds ratio (OR) should not be used, as it is only applicable for rare events. Using OR in our study may lead to overestimation of the effect sizes. This study was a cross-sectional study and the prevalence of co-utilization of ORS and zinc was greater than 10%, and if we report the odds ratio, it could overestimate the association between co-utilization of ORS and zinc and the independent variables. As the prevalence ratio is the best measure of association in these situations, multilevel Poisson regression analysis with robust variance was fitted to identify predictors of zinc and ORS co-

For peer review only

utilization among under-five children with diarrhoea. In the bi-variable multilevel Poisson regression analysis, variables having a p-value<0.2 were considered for the multivariable analysis.

For the multilevel Poisson regression analysis, four models were constructed. The first model aimed to ascertain the degree of cluster variation in the co-utilization of zinc and ORS and it was a null model devoid of explanatory factors. The second model was fitted with individual-level variables, the third with community-level variables, and the fourth with both individual and community-level variables at the same time. Because the models were nested, the deviance -2Log-Likelihood Ratio (LLR) was used to compare them, and the model with the lowest deviance was the best-fitted model for the data. Finally, the 95% confidence interval (CI) for the adjusted prevalence ratio (APR) was determined, and factors in the multivariable analysis with a p-value of < 0.05 were deemed significant predictors of the co-utilization of zinc and ORS among under-five children with diarrhoea.

Patient and public involvement

In this study, the study participants and/or the public were not directly involved in the design, conduct, reporting, and dissemination of this work.

Results

Socio-demographic characteristics

A total weighted sample of 16,850 under-five children who had diarrhea within 2 weeks preceding the survey were included in this study. More than half, 8,965 (53.20%) of the children were males while the vast majority (79.10%) of them resided in rural areas. The majority, 7,778 (46.16%)

For peer review only

193 were from poor households and 3,666 (21.76%) had mothers who did not have a formal education.

194 Only 818 (5.72%) were covered by health insurance (*Table 1*).

195 Table 1: Socio-demographic and economic characteristics of study participants in East Africa

196 from 2011 to 2022.

Variable	Weighted frequency	Percent
**Individual level variables**		
**Sex of the child**		
**Male child**	8,965	53.20
**Female child**	7,885	46.80
**Age of the mother**		
**15-19**	1,433	8.51
**20-24**	4,715	27.98
**25-29**	4,545	26.97
**30-34**	3,228	19.16
**35-39**	1,965	11.66
**40-44**	757	4.49
**45-49**	206	1.22
**Marital status**		
**Never in union**	965	5.73
**Married/ living together**	14,160	84.03
**Divorced/widowed/ separated**	1,725	10.24

For peer review only

**Maternal education**		
**No formal education**	3,666	21.76
**Primary**	8,544	50.71
**Secondary**	3,942	23.40
**Higher**	697	4.14
**Father's education (n=14,164)**		
**No formal education**	2,667	18.83
**Primary**	6,842	48.31
**Secondary**	3,657	25.82
**Higher**	997	7.04
**Maternal current working status**		
**Not Employed**	5,747	34.11
**Employed**	11,103	65.89
**Sex of household head**		
**Male household head**	12,778	75.84
**Female household head**	4,072	24.16
**Household wealth status**		
**Poor**	7,778	46.16
**Middle**	3,218	19.10
**Rich**	5,854	34.74
**Covered by health insurance (n=14,291)**		
**No**	13,472	94.28

For peer review only

**Yes**	818	5.72
**Media exposure**		
**No**	5,959	35.36
**Yes**	10,891	64.64
**Community level variables**		
**Distance to health facility (n=15,498)**		
**Big problem**	6,926	44.69
**Not a big problem**	8,572	55.31
**Residence**		
**Urban**	3,521	20.90
**Rural**	13,329	79.10
**Community educational status**		
**Low**	2,996	17.78
**High**	13,854	82.22
**Community poverty level**		
**Low**	7,695	45.67
**High**	9,155	54.33

The prevalence of ORS and Zinc co-utilization for diarrhea treatment

The pooled prevalence of co-utilization of ORS and zinc for the treatment of diarrhea among under-five children in East African countries was 53.27% with a 95% CI (52.54%, 54.01%). In

For peer review only

East African countries, the prevalence of ORS and zinc co-utilization ranges from 24.36% (95% CI: 21.85, 26.86) in Madagascar to 69.32% (95% CI: 67.76, 70.87) in Malawi (Figure 1).

Figure 1: Forest plot of pooled prevalence of co-utilization of ORS and zinc for management of diarrhoea among under-five children in East Africa from 2011 to 2022.

Random effect and model comparison

To evaluate the random or clustering effect using the intraclass correlation coefficient (ICC), we used deviance for model comparison. The null model's ICC of 0.145 indicated that unmeasured or unmeasurable factors (random effects) accounted for about 14.5% of the variance in the co-utilization of zinc and ORS. In terms of comparing models, the best-fitted model was the final model, which had a lower deviance (Table 2).

Table 2: Random effect and model comparison for factors associated with co-utilization of ORS and zinc for management of diarrhoea among under-five children in East Africa from 2011 to 2022.

	**Null model**	**Model 1**	**Model 2**	**Model 3**
**ICC**	14.56%	15.31%	15.01%	15.01%
**Log-likelihood**	-14402.21	-14369.22	-14398.24	-14367.59
**Deviance**	28,804.42	28,738.44	28,796.48	28,735.18

ICC, intraclass correlation coefficient

Factors associated with co-utilization of ORS and Zinc for under-five diarrhea treatment

A multilevel bi-variable analysis was done to assess the crude relationship between the independent variables and ORS-zinc co-utilization. Among the variables included in the bi-variable analysis only maternal age, maternal education status, wealth index, maternal employment

For peer review only

status, media exposure, community-level illiteracy, and community-level poverty showed a statistically significant association with ORS and zinc co-utilization at a *p*-value of < 0.20.

In the final model maternal age, maternal education status, wealth index, and media exposure were significantly associated with ORS and zinc co-utilization (p-value ≤ 0.05). The prevalence of ORS and zinc co-utilization was 1.14 (APR=1.14, 95%CI: 1.07, 1.21), 1.13 (APR=1.13, 95%CI: 1.06, 1.21), and 1.09 (APR=1.09, 95%CI: 1.02, 1.16) times higher in children whose mothers were aged 20-24, 25-29, and 30-34, respectively in comparison to mother aged 15-19. Being the child of a woman with a primary, secondary, or higher level of education raises the prevalence of co-utilization by 1.15, 1.08, and 1.19, respectively. Compared to children from poor household wealth index, those children from rich wealth index had a 4 % higher prevalence of ORS and zinc co-utilization. The prevalence of ORS and zinc co-utilization among children whose mothers had media exposure was 1.04 times higher as compared to their counterparts (APR = 1.04, 95% CI :1.01, 1.08) (*Table 3*).

Table 3: Factors associated with co-utilization of ORS and zinc for management of diarrhoea among under-five children in East Africa from 2011 to 2022.

**Variable**	**ORS and Zinc Co-utilization**		**CPR (95%CI)**	**APR (95%CI)**	**P-value**
**Individual level variables**	**No (%)**	**Yes (%)**			
**Age of the mother**					
**15-19**	746 (52.03)	687 (47.97)	1	1	
**20-24**	2,106 (44.67)	2,609 (55.33)	1.13 (1.06, 1.2)	1.14 (1.07, 1.2)*	< 0.01
**25-29**	2,026 (44.57)	2,519 (55.43)	1.12 (1.05, 1.2)	1.13 (1.06,1.2)*	< 0.01

For peer review only - http://bmjopen.bmj.com/site/about/guidelines.xhtml

**30-34**	1,573 (48.73)	1,654 (51.27)	1.06 (0.99, 1.1)	1.09 (1.02, 1.2)*	< 0.01
**35-39**	982 (49.99)	982 (50.01)	1.01 (0.94, 1.1)	1.04 (0.97, 1.1)	0.256
**40-44**	400 (52.90)	357 (47.10)	0.95 (0.87, 1.0)	1.00 (0.92, 1.1)	0.929
**45-49**	106 (51.32)	100 (48.68)	0.98 (0.85, 1.1)	1.05 (0.91, 1.2)	0.530
**Maternal educational status**					
**No formal education**	2,004 (54.67)	1,662 (45.33)	1	1	
**Primary**	3,873 (45.33)	4,671 (54.67)	1.17 (1.12, 1.2)	1.15 (1.09, 1.2)*	< 0.01
**Secondary**	1,806 (45.81)	2,136 (54.19)	1.14 (1.08, 1.2)	1.08 (1.02, 1.1)*	< 0.08
**Higher**	256 (36.75)	440 (63.25)	1.28 (1.19, 1.4)	1.19 (1.10, 1.3)*	< 0.01
**Household wealth index**					
**Poor**	3,819 (49.10)	3,958 (50.90)	1	1	
**Middle**	1,511 (46.96)	1,707 (53.04)	1.05 (1.01, 1.1)	1.03 (0.99, 1.1)	0.137
**Rich**	2,609 (44.57)	3,245 (55.43)	1.07 (1.03, 1.1)	1.04 (1.01, 1.1)*	0.042
**Maternal employment status**					
**Not employed**	2,769 (48.19)	2,977 (51.81)	1	1	
**Employed**	5,170 (46.57)	5,933 (53.43)	0.98 (0.95, 1.1)	0.97 (0.94, 1.04)	0.08
**Media exposure**					
**No**	3,022 (50.72)	2,936 (49.28)	1	1	
**Yes**	4,917 (45.15)	5,974 (54.85)	1.08 (1.04, 1.1)	1.04 (1.01, 1.1)*	0.046
**Community illiteracy level**					

**Low**	1,287 (42.96)	1,709 (57.04)	1	1	
**High**	6,653 (48.02)	7,201 (51.98)	0.92 (0.89, 0.9)	0.96 (0.92, 1.03)	0.065
**Community poverty level**					
**Low**	3,641 (47.32)	4,053 (52.68)	1	1	
**High**	4,298 (46.95)	4,857 (53.05)	0.99 (0.97, 1.0)	1.03 (0.99, 1.1)	0.095

233 *p-value ≤ 0.05, CPR: Crude Prevalence Ratio, APR: Adjusted Prevalence Ratio

234 Discussion

235 This study aimed to assess the factors associated with the co-use of ORS and zinc for the treatment
236 of diarrhoea in under-five children across East African countries. In our study, the co-use of ORS
237 and zinc was associated with the mother's age, her educational level, family's financial status, and
238 media exposure.

239 This study mentioned maternal age above twenty is associated with co-utilization of ORS and zinc
240 and this finding is supported by different study conducted in Nigeria (26). The possible reason for
241 this finding is older mothers were more likely than younger mothers to be knowledgeable about
242 pediatric diarrhoea and how to treat it, younger mothers do not have as much exposure as older
243 mothers about child diarrhoea and its management as maternal age can have impact on their
244 knowledge about and how to treat it (27). This study found that maternal educational status was
245 associated with the usage of ORS with zinc to treat under-five diarrheal disease and this finding is
246 supported by studies conducted in Pakistan (28), India (29), Nigeria (23), Cameron (30), Kenya
247 (31), and Ethiopia (21, 25, 32-34). This may be because educated women are more likely to take
248 sick children to the medical facility and a woman with education is more likely than a mother

For peer review only

without education to take her ill kid for follow-up as well as to take the ordered medication as per recommended. In addition to the above reason, educated mothers have a better awareness regarding the health problems of their kids than a mother without education (35, 36).

Our study mentioned that media exposure was one of the factors associated with diarrheal treatment using ORS with zinc. This finding is in line with studies conducted in India (29), Bangladesh (37), Ghana (38), and Ethiopia (25, 34). This may be due to the media's important role in increasing the mother's or caregiver's understanding and awareness of the best ways to treat diarrhoea in children (37).

As this study finds, a family's wealth status affects the usage of ORS with zinc. This finding is supported by different studies conducted in India (39), and Ethiopia (25). This is due to the fact that low-income households in low and middle-income countries (LMICs), such as those in Africa, particularly in East Africa, cannot afford the expenses of healthcare services, including ORS and zinc. Thus, when children from low-income households get diarrhoea, they might not receive ORS with zinc (40).

Our study was based on nationally representative large data and used appropriate statistical analysis techniques. Despite the aforementioned strengths, the measure of zinc utilization practice was based on the mother's recall, which could lead to recall bias. Second, the DHS survey lacks information on zinc availability and the role of diet in zinc intake. As a result, we are unable to evaluate these factors. Additionally Some East African countries are not included in the DHS program. Furthermore, due to the cross-sectional nature of the data, a clear temporality (cause and effect relationship) between the dependent and independent variables was not established.

For peer review only

Conclusion

In this study, media exposure, rich household wealth status, maternal education, and maternal age over twenty were associated factors for ORS and zinc co-use. To raise the prevalence of ORS and zinc co-utilization for the successful treatment of diarrhoea in children under the age of five, it is advised to increase media exposure, maternal education, and support the household wealth index. Using media as tool to advertise the importance of the co-utilization of ORS and zinc for all community will increase and boost the utilization of the product. Empowering women through education also will raise the co-utilization of ORS and zinc. In addition, policies and intervention programs that aim to stop unnecessary deaths of children under the age of five should be prioritized by preventing adolescent pregnancy.

Abbreviations: aPR, Adjusted Prevalence Ratio; CI, Confidence Interval; uPR, Unadjusted Prevalence Ratio; DHS, Demographic and Health Survey; ICC, Intraclass Correlation Coefficient; LL: Log-likelihood; UNICEF: United Nations Children's Fund

Competing interests

The authors declare that they have no competing interests.

Authors' contributions

Conceptualization: Bruck Tesfaye Legesse, Beminate Lemma Seifu

Data curation: Bruck Tesfaye Legesse, Beminate Lemma Seifu

Formal analysis: Beminate Lemma Seifu, Bruck Tesfaye Legesse

Investigation: Bruck Tesfaye Legesse, Beminate Lemma Seifu

For peer review only

Methodology: Beminate Lemma Seifu, Bruck Tesfaye Legesse

Software: Bruck Tesfaye Legesse, Beminate Lemma Seifu

Validation: Beminate Lemma Seifu, Bruck Tesfaye Legesse, Wubet Tazeb Wondie, Gezahagn Demsu Gedefaw, Yakob Tadese Workineh

Writing – original draft: Bruck Tesfaye Legesse, Beminate Lemma Seifu

Writing – review & editing: Beminate Lemma Seifu, Bruck Tesfaye Legesse, Wubet Tazeb Wondie, Gezahagn Demsu Gedefaw, Yakob Tadese Workineh

Acknowledgments

We express our gratitude to the MEASURE DHS for providing the study's dataset.

Funding

No fund was received for this work.

Availability of data and materials

All the data used for the study are available through an online request to http://www.dhsprogram.com

Consent for publication

Not applicable.

Supporting information files

Supporting file 1: STROBE Statement—Checklist of items that should be included in reports of cross-sectional studies

For peer review only

Ethical consideration

Through an online request to http://www.dhsprogram.com, permission to access the measure demographic and health survey data used in this study was obtained. Data from the official program database are accessible to the public.

Reference

1. Guarino A, Dupont C, Gorelov AV, Gottrand F, Lee JK, Lin Z, et al. The management of acute diarrhea in children in developed and developing areas: from evidence base to clinical practice. 2012;13(1):17-26.

2. Almasi A, Zangeneh A, Ziapour A, Saeidi S, Teimouri R, Ahmadi T, et al. Investigating Global Spatial Patterns of Diarrhea-Related Mortality in Children Under Five. 2022;10:861629.

3. Mokomane M, Kasvosve I, Melo Ed, Pernica JM, Goldfarb DMJTaiid. The global problem of childhood diarrhoeal diseases: emerging strategies in prevention and management. 2018;5(1):29-43.

4. Rahman AE, Moinuddin M, Molla M, Worku A, Hurt L, Kirkwood B, et al. Childhood diarrhoeal deaths in seven low-and middle-income countries. 2014;92(9):664.

5. Acácio S, Mandomando I, Nhampossa T, Quintó L, Vubil D, Sacoor C, et al. Risk factors for death among children 0–59 months of age with moderate-to-severe diarrhea in Manhiça district, southern Mozambique. 2019;19(1):1-14.

6. CSA IJAA, Ethiopia, Calverton M, USA: Central Statistical Agency, International I. Ethiopia demographic and health survey 2011. 2012;430.

7. O'Reilly CE, Jaron P, Ochieng B, Nyaguara A, Tate JE, Parsons MB, et al. Risk factors for death among children less than 5 years old hospitalized with diarrhea in rural western Kenya, 2005–2007: a cohort study. 2012;9(7):e1001256.

8. Lee BX, Kjaerulf F, Turner S, Cohen L, Donnelly PD, Muggah R, et al. Transforming our world: implementing the 2030 agenda through sustainable development goal indicators. 2016;37:13-31.

9. Qazi S, Aboubaker S, MacLean R, Fontaine O, Mantel C, Goodman T, et al. Ending preventable child deaths from pneumonia and diarrhoea by 2025. Development of the integrated

For peer review only

Global Action Plan for the Prevention and Control of Pneumonia and Diarrhoea. 2015;100(Suppl 1):S23-S8.

10. Black R, Fontaine O, Lamberti L, Bhan M, Huicho L, El Arifeen S, et al. Drivers of the reduction in childhood diarrhea mortality 1980-2015 and interventions to eliminate preventable diarrhea deaths by 2030. 2019;9(2).

11. Hill S, Yang A, Bero L. Priority medicines for maternal and child health: a global survey of national essential medicines lists. PloS one. 2012;7(5):e38055.

12. Roche M, García Meza R, Vossenaar M. An intervention to Co-package zinc and oral rehydration salts (ORS) improves health provider prescription and maternal adherence to WHO-recommended diarrhea treatment in Western Guatemala. The FASEB Journal. 2015;29:902.23.

13. Borapich D, Warsh M, Borapich D, Warsh M. Improving child health in Cambodia: social marketing of diarrhea treatment kit, results of a pilot project. Cases in Public Health Communication & Marketing. 2010;4(4):22.

14. Barffour MA, Hinnouho G-M, Wessells KR, Kounnavong S, Ratsavong K, Sitthideth D, et al. Effects of therapeutic zinc supplementation for diarrhea and two preventive zinc supplementation regimens on the incidence and duration of diarrhea and acute respiratory tract infections in rural Laotian children: A randomized controlled trial. Journal of global health. 2020;10(1).

15. Lazzerini M, Wanzira H. Oral zinc for treating diarrhoea in children. Cochrane database of systematic reviews. 2016(12).

16. Organization WH. Ending preventable child deaths from pneumonia and diarrhoea by 2025: the integrated global action plan for pneumonia and diarrhoea (GAPPD). 2013.

17. Alam J, Nuzhat S, Billal SM, Ahmed T, Khan AI, Hossain MI. Nutritional Profiles and Zinc Supplementation among Children with Diarrhea in Bangladesh. The American journal of tropical medicine and hygiene. 2023;108(4):837-43.

18. Gona PN, Gona CM, Chikwasha V, Haruzivishe C, Rao SR, Mapoma CC. Oral rehydration solution coverage in under 5 children with diarrhea: a tri-country, subnational, cross-sectional comparative analysis of two demographic health surveys cycles. BMC Public Health. 2020;20(1):1-15.

For peer review only

19. Mohamed SOO, Alawad MOA, Ahmed AAM, Mahmoud AAAJBRN. Access to oral rehydration solution and zinc supplementation for treatment of childhood diarrhoeal diseases in Sudan. BMC Research Notes. 2020;13.

20. Lam F, Wentworth L, Cherutich P, Migiro S, Abdala K, Musyoka M, et al. An evaluation of a national oral rehydration solution and zinc scale-up program in Kenya between 2011 and 2016. 2019;9(1).

21. Kassa SF, Alemu TG, Techane MA, Wubneh CA, Assimamaw NT, Belay GM, et al. The co-utilization of oral rehydration solution and zinc for treating diarrhea and its associated factors among under-five children in Ethiopia: further analysis of EDHS 2016. 2022:1713-21.

22. Lam F, Kirchhoffer D, Buluma DM, Kabunga L, Wamala-Mucheri PN, Schroder K, et al. A retrospective mixed-methods evaluation of a national ORS and zinc scale-up program in Uganda between 2011 and 2016. 2019;9(1).

23. Gwarzo GJNJoP. Mothers' awareness and use of zinc in under-five children with diarrhoea in North-Western Nigeria. 2018;45(2):81-5.

24. Ajayi DJAoB, Medicine A. Determinants of Oral Rehydration Solution and Zinc Use Among Under-Five Children for The Management of Diarrhea in Abeokuta, Nigeria. 2019;7(1):35–9-–9.

25. Negesse Y, Fetene Abebe G, Addisu A, Setegn Alie M, Alemayehu DJFiPH. The magnitude of oral rehydration salt utilization in diarrhea hot spot regions of Ethiopia and its associated factors among under-five children: A multilevel analysis based on Bayesian approach. 2022;10:960627.

26. Okafor IP, Akinyemi OT, Wika-Kobani BN, Olubodun T, Eze UT. Childhood diarrhoea: a cross-sectional survey on maternal knowledge, hygienic practices and use of oral zinc for home management in a Nigerian community. The Pan African medical journal. 2022;42:123.

27. Okoh BA, Alex-Hart BA. Home management of diarrhoea by caregivers presenting at the diarrhoea training unit of a tertiary hospital in southern Nigeria. British Journal of Medicine and Medical Research. 2014;4(35):5524-40.

28. Duggal MN, Bari A, Mansoor A, Saeed S, Rizwan W, Nayar JJJTJotPMA. Awareness of quantity of ORS and Zinc among mothers of under five children with diarrhoea at a tertiary care Centre Lahore. 2021;71(3):921-4.

For peer review only

29. Lam F, Pro G, Agrawal S, Shastri VD, Wentworth L, Stanley M, et al. Effect of enhanced detailing and mass media on community use of oral rehydration salts and zinc during a scale-up program in Gujarat and Uttar Pradesh. 2019;9(1).

30. Essomba NE, Koum DK, Adiogo D, Ngwe M, Coppieters YJMmj. Use of oral rehydration therapy in the treatment of childhood diarrhoea in Douala, Cameroon. 2015;27(2):60-4.

31. Njeri G, Muriithi MJESJ. Household choice of diarrhea treatments for children under the age of five in Kenya: evidence from The Kenya Demographic and Health Survey 2008-09. 2013;9(6):77-91.

32. Ayele E, Tasew H, Mariye T, Teklay G, Alemayhu T, Mesfin FJM. Zinc utilization and associated factors among under-five children having acute diarrhea in Kebri-dehar Town, Somali Region, Ethiopia-2017. 2020;4(1):15-9.

33. Terefa DR, Shama AT, Kenea AKJBo. Sociodemographic and institutional determinants of zinc bundled with oral rehydration salt utilisation among under-five children with diarrhoeal diseases in East Wallaga zone, western Ethiopia: a community-based cross-sectional study. 2023;13(5):e070203.

34. Teshale AB, Liyew AM, Tesema GAJBPH. Factors associated with zinc utilization for the management of diarrhea in under-five children in Ethiopia. 2020;20:1-7.

35. Adane M, Mengistie B, Mulat W, Kloos H, Medhin GJJoH, Population, Nutrition. Utilization of health facilities and predictors of health-seeking behavior for under-five children with acute diarrhea in slums of Addis Ababa, Ethiopia: a community-based cross-sectional study. 2017;36(1):1-12.

36. Apuleni G, Jacobs C, Musonda P. Predictors of health seeking behaviours for common childhood illnesses in poor resource settings in Zambia, a community cross sectional study. Frontiers in Public Health. 2021;9:569569.

37. Alam Z, Higuchi M, Sarker MAB, Hamajima NJNjoms. Mass media exposure and childhood diarrhea: a secondary analysis of the 2011 Bangladesh demographic and health survey. 2019;81(1):31.

38. El-Khoury M, Banke K, Sloane PJGHS, Practice. Improved childhood diarrhea treatment practices in Ghana: a pre-post evaluation of a comprehensive private-sector program. 2016;4(2):264-75.

For peer review only

39. Bawankule R, Singh A, Kumar K, Pedgaonkar SJBph. Disposal of children's stools and its association with childhood diarrhea in India. 2017;17:1-9.

40. Braimoh T, Danat I, Abubakar M, Ajeroh O, Stanley M, Wiwa O, et al. Private health care market shaping and changes in inequities in childhood diarrhoea treatment coverage: evidence from the analysis of baseline and endline surveys of an ORS and zinc scale-up program in Nigeria. 2021;20:1-12.

For peer review only

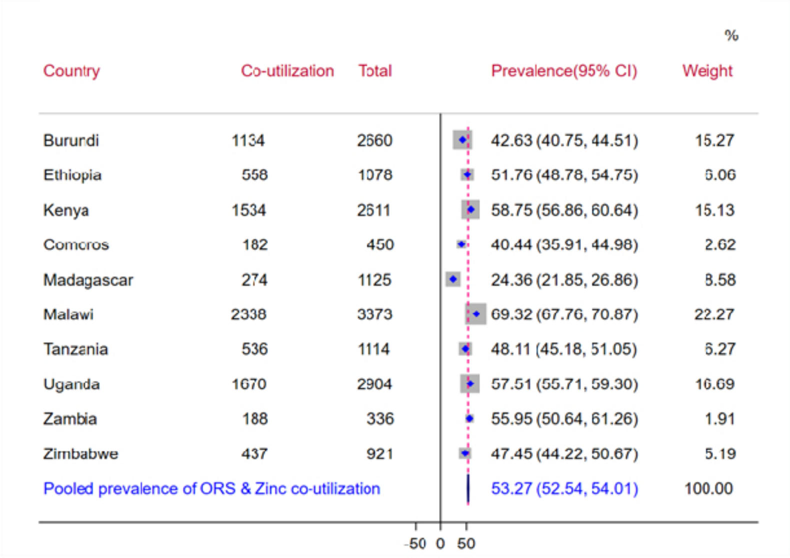

204x120mm (96 x 96 DPI)

STROBE Statement—Checklist of items that should be included in reports of ***cross-sectional studies***

	Item No	Recommendation	Page No
**Title and abstract**	1	(*a*) Indicate the study's design with a commonly used term in the title or the abstract	1
		(*b*) Provide in the abstract an informative and balanced summary of what was done and what was found	2
**Introduction**			
Background/rationale	2	Explain the scientific background and rationale for the investigation being reported	2
Objectives	3	State specific objectives, including any prespecified hypotheses	1, 5
**Methods**			
Study design	4	Present key elements of study design early in the paper	5, 6
Setting	5	Describe the setting, locations, and relevant dates, including periods of recruitment, exposure, follow-up, and data collection	5
Participants	6	(*a*) Give the eligibility criteria, and the sources and methods of selection of participants	5
Variables	7	Clearly define all outcomes, exposures, predictors, potential confounders, and effect modifiers. Give diagnostic criteria, if applicable	6, 7
Data sources/ measurement	8*	For each variable of interest, give sources of data and details of methods of assessment (measurement). Describe comparability of assessment methods if there is more than one group	*7*
Bias	9	Describe any efforts to address potential sources of bias	7
Study size	10	Explain how the study size was arrived at	6, 7
Quantitative variables	11	Explain how quantitative variables were handled in the analyses. If applicable, describe which groupings were chosen and why	
Statistical methods	12	(*a*) Describe all statistical methods, including those used to control for confounding	7
		(*b*) Describe any methods used to examine subgroups and interactions	
		(*c*) Explain how missing data were addressed	
		(*d*) If applicable, describe analytical methods taking account of sampling strategy	
		(*e*) Describe any sensitivity analyses	
**Results**			
Participants	13*	(a) Report numbers of individuals at each stage of study—eg numbers potentially eligible, examined for eligibility, confirmed eligible, included in the study, completing follow-up, and analysed	5
		(b) Give reasons for non-participation at each stage	
		(c) Consider use of a flow diagram	
Descriptive data	14*	(a) Give characteristics of study participants (eg demographic, clinical, social) and information on exposures and potential confounders	8, 9
		(b) Indicate number of participants with missing data for each variable of interest	
Outcome data	15*	Report numbers of outcome events or summary measures	10
Main results	16	(*a*) Give unadjusted estimates and, if applicable, confounder-adjusted estimates and their precision (eg, 95% confidence interval). Make clear which confounders were adjusted for and why they were included	10, 11

For peer review only

		(*b*) Report category boundaries when continuous variables were categorized	
		(*c*) If relevant, consider translating estimates of relative risk into absolute risk for a meaningful time period	
Other analyses	17	Report other analyses done—eg analyses of subgroups and interactions, and sensitivity analyses	
**Discussion**			
Key results	18	Summarise key results with reference to study objectives	11
Limitations	19	Discuss limitations of the study, taking into account sources of potential bias or imprecision. Discuss both direction and magnitude of any potential bias	2
Interpretation	20	Give a cautious overall interpretation of results considering objectives, limitations, multiplicity of analyses, results from similar studies, and other relevant evidence	
Generalisability	21	Discuss the generalisability (external validity) of the study results	12
**Other information**			
Funding	22	Give the source of funding and the role of the funders for the present study and, if applicable, for the original study on which the present article is based	15

*Give information separately for exposed and unexposed groups.

Note: An Explanation and Elaboration article discusses each checklist item and gives methodological background and published examples of transparent reporting. The STROBE checklist is best used in conjunction with this article (freely available on the Web sites of PLoS Medicine at http://www.plosmedicine.org/, Annals of Internal Medicine at http://www.annals.org/, and Epidemiology at http://www.epidem.com/). Information on the STROBE Initiative is available at www.strobe-statement.org.

For peer review only
